# Long noncoding RNA BS-DRL1 modulates the DNA damage response and genome stability by interacting with HMGB1 in neurons

**DOI:** 10.1038/s41467-021-24236-z

**Published:** 2021-07-01

**Authors:** Min-Min Lou, Xiao-Qiang Tang, Guang-Ming Wang, Jia He, Fang Luo, Ming-Feng Guan, Fei Wang, Huan Zou, Jun-Ying Wang, Qun Zhang, Ming-Jian Xu, Qi-Li Shi, Li-Bing Shen, Guo-Ming Ma, Yi Wu, Yao-Yang Zhang, Ai-bin Liang, Ting-Hua Wang, Liu-Lin Xiong, Jian Wang, Jun Xu, Wen-Yuan Wang

**Affiliations:** 1 Interdisciplinary Research Center on Biology and Chemistry, Shanghai Institute of Organic Chemistry, Chinese academy of Science, Shanghai, 200032 China; 2 University of Chinese Academy of Sciences, Beijing, China; 3 East Hospital, Tongji University School of Medicine, Shanghai, China; 4 Department of Hematology, Tongji Hospital of Tongji University School of Medicine, Shanghai, China; 5 Postdoctoral Station of Clinical Medicine, Shanghai Tongji Hospital, Tongji University School of Medicine, Shanghai, China; 6 Department of Rehabilitation Medicine, Huashan Hospital, Fudan University, Shanghai, China; 7 Animal Center of Zoology, Institute of Neuroscience, Kunming medical University, Kunming, China; 8 Research Center for Aging and Medicine, Huashan Hospital, Fudan University, Jing’an District, Shanghai, China

**Keywords:** DNA damage and repair, Long non-coding RNAs, Cellular neuroscience

## Abstract

Long noncoding RNAs (lncRNAs) are known to regulate DNA damage response (DDR) and genome stability in proliferative cells. However, it remains unknown whether lncRNAs are involved in these vital biological processes in post-mitotic neurons. Here, we report and characterize a lncRNA, termed Brain Specific DNA-damage Related lncRNA1 (BS-DRL1), in the central nervous system. BS-DRL1 is a brain-specific lncRNA and depletion of BS-DRL1 in neurons leads to impaired DDR upon etoposide treatment in vitro. Mechanistically, BS-DRL1 interacts with HMGB1, a chromatin protein that is important for genome stability, and is essential for the assembly of HMGB1 on chromatin. BS-DRL1 mediated DDR exhibits cell-type specificity in the cortex and cerebellum in gamma-irradiated mice and BS-DRL1 knockout mice show impaired motor function and concomitant purkinje cell degeneration. Our study extends the understanding of lncRNAs in DDR and genome stability and implies a protective role of lncRNA against neurodegeneration.

## Abstract (web summary)

Long noncoding RNAs (lncRNAs) are known to regulate the DNA damage response (DDR), however their role in the brain is less well studied. Here, the authors demonstrate a neuron-specific role for Brain Specific DNA-damage Related lncRNA1 (BS-DRL1) and show BS-DRL1 modulates DDR by interacting with HMGB1 in a cell-type specific manner.

## Introduction

lncRNAs are arbitrarily defined as a group of transcripts longer than 200 nucleotides that are generally not translated into proteins, but are functionally involved in many physiological and pathological processes, including development, aging, and diseases through modulating the activity of associated proteins or mRNAs, organizing subnuclear structure, and mediating chromosomal interactions^1–3^. However, due to their general low abundance and modest evolutionary conservation compared to protein-coding genes, the biological roles and the related molecular mechanisms for the majority of lncRNAs remain unexplored^4^. Recent studies reveal that many lncRNAs are crucial for DNA damage response (DDR), DNA repair, and genome stability in cancer cells^5–7^. Although the brain is specifically enriched with numerous non-coding RNAs, it remains unknown whether they play a role in DDR and repair, which is increasingly recognized as an indispensable factor for neurodegeneration and brain aging.

The DNA damage and genome instability incurred by either endogenous cellular metabolic products, such as 8-oxo-dG, or environmental chemicals can lead to the accumulation of unrepaired or erroneously-repaired DNA breaks, and the alteration of chromatin organization. Together, these destructive changes can profoundly affect the integrity of neuronal functions and therefore contribute to brain aging and to neurodegeneration^8^. For example, the accumulation of oxidative lesions in the promoter regions of genes that are responsible for critical neuronal functions, such as synaptic plasticity, learning, and memory, leads to the downregulation of gene expression after age 40^9^. Furthermore, it has been reported that 13–41% of neurons from the human frontal cortex harbor copy number variations (CNVs)^10^. Although the origin and precise consequence of this somatic mosaicism in the human brain are currently unclear, there is no doubt that it is related to defects with DNA repair.

In addition to normal brain aging, DNA damage and genome instability have also been linked to age-related neurodegenerative diseases, such as Alzheimer’s disease (AD), Parkinson’s disease (PD), and amyotrophic lateral sclerosis (ALS)^8^. For instance, accumulation of damaged DNA, elevated levels of oxidative lesions, and reduced expression of DNA repair proteins have been reported in AD and ALS patients compared to age-matched controls^11,12^. Moreover, a very high level of mitochondrial DNA damage has been detected in substantia nigra neurons of aged people^13^.

HMGB1, a conserved non-histone chromatin-associated protein with important roles in regulating the tertiary structure of chromatin, influences a broad range of nuclear functions including transcription, DNA repair, and genome stability^14,15^. HMGB1 participates in multiple types of DNA repair, such as mismatch repair, nucleotide excision repair, base excision repair, and the non-homologous end joining^16^. The function of HMGB1 in DDR is mediated by its ability to bind to damaged DNA and interact with repair enzymes^17^. A number of studies have implicated HMGB1 in various diseases. For example, the reduced HMGB1 levels causes increased mitochondrial DNA damage, whereas increased HMGB1 levels enhances the repair of mitochondrial DNA damage and extends the lifespan of mutant ataxin-1 knock-in mice^18^. HMGB1 also suppresses genotoxic stress in polyglutamine diseases^19^.

In this study, we have discovered a brain-specific lncRNA, named herein as BS-DRL1, and characterized its functions as an important regulator of DDR and genome stability in neurons both in vitro and in vivo. We identified that BS-DRL1 interact with HMGB1 and is functionally essential for the assembly of the HMBG1 on chromatin upon DNA damage. Notably, BS-DRL1 KO mice exhibited cell type-specific DDR in different brain regions after gamma-irradiation (e.g., NeuN-positive cells in cortex and NeuN-negative cells in cerebellum), impaired locomotion ability, and Purkinje cell degeneration. Our results revealed a previously unknown lncRNA-dependent mechanism that is essential for maintaining genomic stability in the brain.

## Results

### Expression and functional characterization of BS-DRL1

**Fig. 1:**
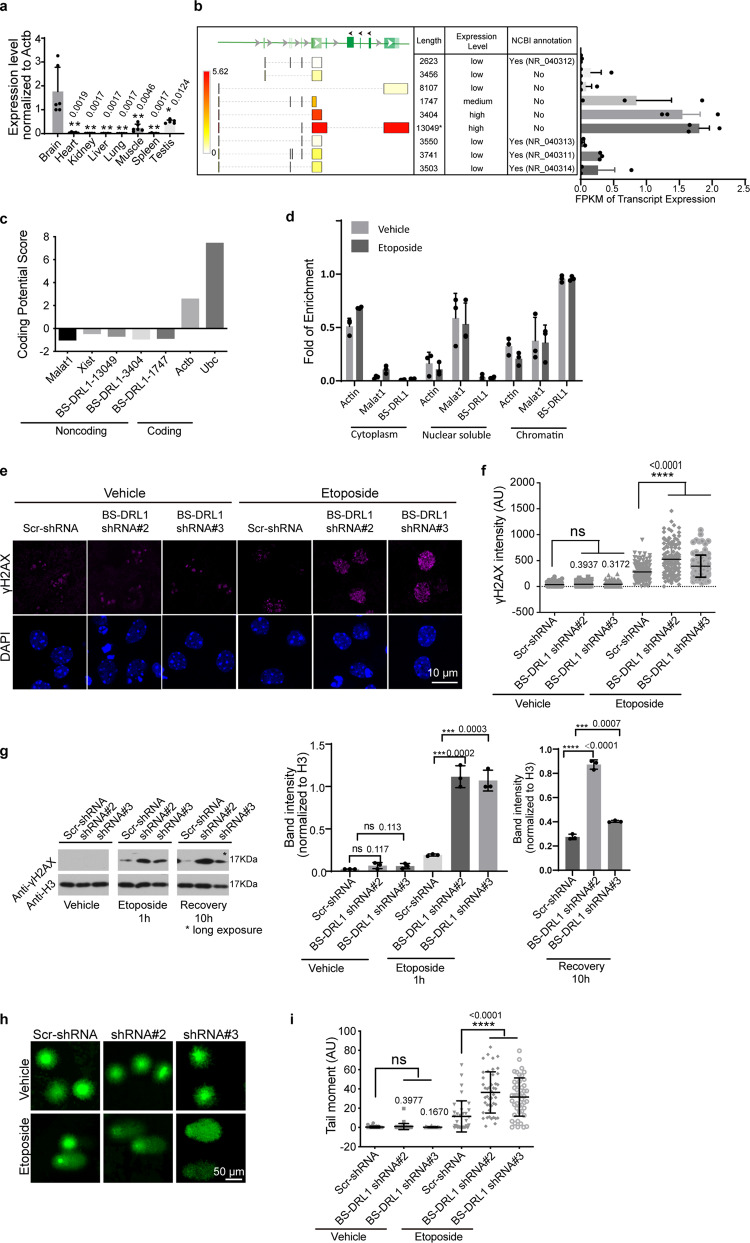
Expression and functional characterization of BS-DRL1. **a** mRNA expression level of BS-DRL1 across different mice tissues (8–10 weeks) was measured by RT-qPCR. Data are presented as mean ± SD (*n* = 6 mice). **b** Schematic illustration of new BS-DRL1 transcripts (left) and their expression levels in the brain based on the mouse brain RNA-Seq dataset (right). *n* = 3 biologically independent samples. Data are presented as mean ± SD. **c** Prediction of the coding potential of three new BS-DRL1 transcripts. Actb(Beta-actin) and Ubc(Ubiquitin C) were used as positive controls, whereas Malat1 and Xist were used as negative controls. **d** Subcellular distribution of BS-DRL1 quantified by fractionation and RT-qPCR. Lysate of ETO-treated neurons were separated into cytoplasm, nuclear soluble and insoluble fractions, from which RNA was extracted and measured by RT-qPCR. *n* = 3 biologically independent samples. Data are presented as mean ± SD. **e**, **f** DNA damage was evaluated by γH2AX staining in neurons transduced with BS-DRL1 shRNA or Scr-shRNAs virus. Scale bar: 10 μm. Data are presented as mean ± SD, *n* = 16 neurons. ****p < 0.0001, ***p < 0.001, ns: not significant. AU: arbitrary units. **g** Western blot analysis of γH2AX level in primary neurons infected with indicated shRNAs virus. Neurons were treated with vehicle or ETO for 1 h, lysed immediately or allowed to recover for 10 h and then lysed for western blot analysis. Data are presented as mean ±  SD, *n* = 3. ***p < 0.001 ****p < 0.0001. **h**, **i** The level of DNA damage in neurons was measured by comet assay. Primary neurons transduced with indicated shRNAs virus were treated with vehicle or ETO for 1 h and harvested for comet assays. Tail moment was analyzed with CaspLab software. Scale bar: 50 μm. Data are presented as mean ± SD, *n* = 37 neurons. ****p < 0.0001, ns not significant, AU arbitrary units.

To investigate the potential role of lncRNAs in DDR and genome stability in the central nervous system (CNS), we examined the lncRNAs that are both evolutionarily-conserved and brain-enriched. By searching literature and public databases, we selected 3 lncRNAs that are highly conserved and whose expression is enriched in our in-house primary neuron RNA-Seq dataset (Supplementary Fig. 1a). In an initial screen to identify lncRNAs involved in DDR, we found that neurons expressing shRNAs against Mir9-3hg (which we renamed as BS-DRL1, Brain specific DNA-damage related lncRNA1) exhibited altered DDR (Supplementary Fig. 1b, c and Fig. 1g), so we focused on this lncRNA in our study. We verified the expression level of BS-DRL1 in different mouse tissues and confirmed its brain specificity (Fig. 1a). When analyzing our RNA-Seq data with primary neurons, we unexpectedly found that in addition to the 4 annotated transcripts, there were 5 new transcripts of BS-DRL1, three of which are highly expressed in the brain (Fig. 1b). We employed RACE (rapid amplification of cDNA ends) to experimentally validate these three new transcripts and determined their final length to be 1747, 3404, and 13049 bp (Fig. 1b and Supplementary Fig. 1d). The longest 13049 bp transcript, which was originally identified by our RNA-seq analysis as a 13665 bp transcript, is most abundantly expressed in the brain. Both bioinformatic analysis^20^ and ribosome profiling^21^ suggested that these newly identified transcripts are non-coding (Fig. 1c and Supplementary Fig. 1e). Furthermore, BS-DRL1 is predominately localized to the chromatin and its distribution is not affected by etoposide (ETO)-induced DNA damage (Fig. 1d).

Histone modification has been successfully used as a proxy for the identification of novel lncRNAs. To investigate chromatin signatures of the BS-DRL1, we generated ChIP-Seq profiling of Pol II, H3K4me3, H3K27ac, and H3K4me1 around the transcription start site (TSS) and the exons of BS-DRL1 from ENCODE^22^ (Supplementary Fig. 1f). We observed that similar to the protein-coding genes, the histone profiles of BS-DRL1 are enriched with H3K4me3, Pol II, and H3K27ac at the promoter region, indicating active transcription. Indeed, we observed that BS-DRL1 is consistently expressed in adult brain tissue with enrichment in the olfactory bulb, cortex, and cerebellum (Supplementary Fig. 2a–e).

To investigate the function of BS-DRL1 in the neuronal DDR, we induced DNA damage in primary cortical neurons (DIV 14) and evaluated the DDR by measuring immunoreactivity for serine 139–phosphorylated histone H2AX (γH2AX), a well-established marker for DNA damage. We found a dramatically increased DDR after ETO treatment in BS-DRL1 knockdown (KD) neurons compared to controls (Fig. 1e, f). In line with the immunostaining data, western blot analysis also detected increased γH2AX in the BS-DRL1 KD neurons treated with ETO (Fig. 1g). To examine whether BS-DRL1 is involved in DNA repair, primary neurons were treated with ETO for 1 h and allowed to recover for 10 h before western blot analysis. We observed that even after a long exposure, the γH2AX signal in scrambled shRNA (Scr-shRNA) infected neurons was almost completely undetectable, whereas γH2AX still maintained a significant level in the BS-DRL1 KD neurons, indicating a deficit in DNA repair (Fig. 1g).

To directly evaluate the level of DNA damage in neurons after BS-DRL1 KD, we performed a single-cell gel electrophoresis assay (also known as comet assay), a sensitive method to assess the integrity of DNA at the single cell level. While vehicle-treated neurons showed few comet tails, ETO-treated neurons expressing BS-DRL1 shRNA showed markedly increased tail moments compared to cells expressing Scr-shRNA (Fig. 1h, i). This indicates the presence of many more DNA breaks in BS-DRL1 KD neurons following ETO treatment.

We noticed that miR-9-3 is located in the first intron region of BS-DRL1, so it is necessary to determine whether the expression of miR-9-3 is affected when manipulating BS-DRL1. Previous studies suggested that the expression of some intronic miRNAs is significantly correlated with their host genes as they use the same transcriptional start sites to initiate their transcription. However, there is increasing evidence that most of the intronic miRNAs have their own independent promoters and that their transcription is independent of their host genes^23,24^. We found that the expression of miR-9-3 was not significantly changed after BS-DRL1 KD, suggesting that transcription of miR-9-3 is independent of BS-DRL1 (Supplementary Fig. 3a–c). Furthermore, the expression of *Polg*, which is located upstream of BS-DRL1, was also unchanged after BS-DRL1 downregulation, suggesting that BS-DRL1 may not function in *cis* (Supplementary Fig. 3d). These results provided additional support that the DDR impairment we observed was indeed mediated by BS-DRL1.

### BS-DRL1 knockout mice exhibit cell-type specific impairment of DDR in the cortex and cerebellum in vivo

**Fig. 2:**
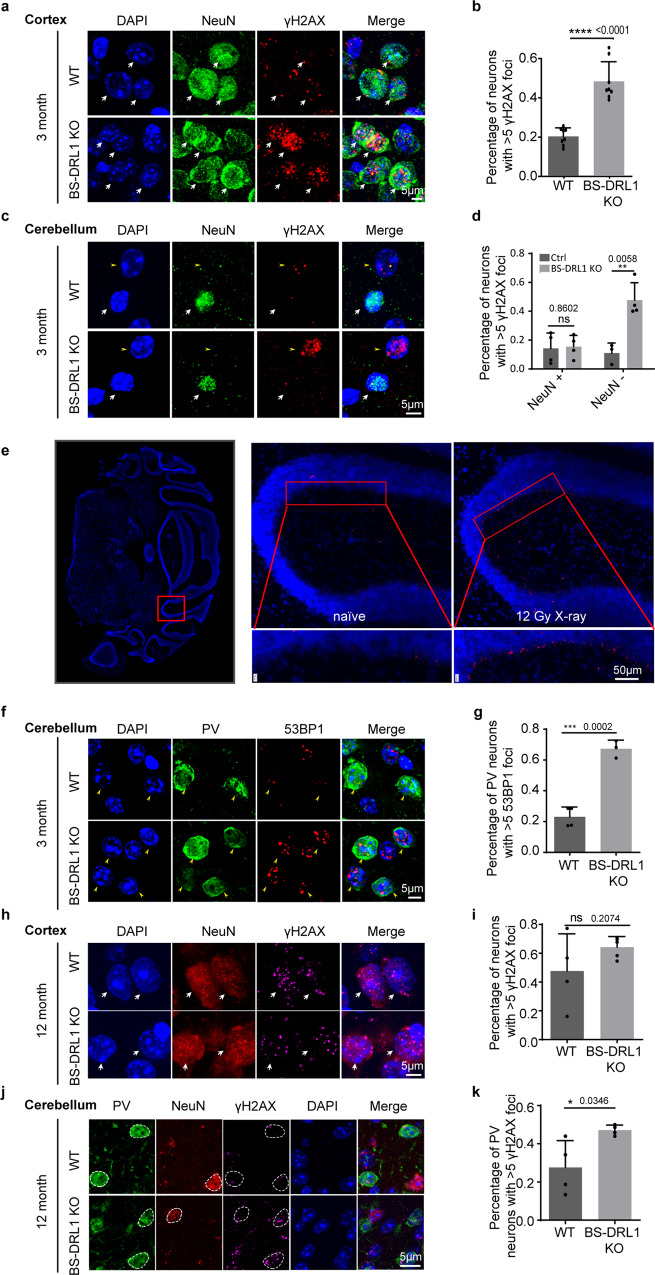
BS-DRL1 KO mice exhibit an impairment in DDR in the cortex and cerebellum in vivo. **a**–**d** Representative images and quantification of DNA damage level in Neurons of gamma-irradiation treated cerebral cortex and cerebellum sections from 3-month-old BS-DRL1 KO mice or littermate controls. Neurons and DNA damage were labeled with NeuN (in green) and γH2AX (in red) antibodies, respectively. 100 neurons from 3 mice were counted for each group. White arrows indicate NeuN+ neurons, yellow arrowheads indicated NeuN− neurons. Scale bar: 5 μm. Data are presented as mean ± SD, ****p < 0.0001, **p < 0.01, ns: not significant. **e** BS-DRL1 in situ with RNAscope probe target 2599-3591 of NR_040311.1. (red). The whole cerebellum (left) is for indicating the location of the enlarged images. Scale bar: 50 μm. Three biologically independent experiments were performed. **f**, **g** Representative images and quantification of DNA damage level in PV Neurons of gamma-irradiation treated cerebellum sections from 3-month-old BS-DRL1 KO mice or littermate controls. Neurons and DNA damage was labeled with parvalbumin (PV, in green) and 53BP1 (in red) antibodies. yellow arrowheads indicate PV neurons. Scale bar: 5 μm. For each group, 100 neurons from 3 mice were counted. Data are presented as mean ± SD, ***p < 0.001. **h**–**k** Representative images and quantification of DNA damage level in Neurons of naive cerebral cortex and cerebellum sections from 12-month-old BS-DRL1 KO mice or littermate controls. Neurons and DNA damage were labeled with NeuN (in red), PV (in green) and γH2AX (in purple) antibodies. White arrows indicated NeuN+ neurons, dotted circles indicated the PV and NeuN neurons used for statistics of γH2AX. Scale bar: 5 μm. For each group, 100 neurons from 3 mice were counted. Data are presented as mean ± SD, *p < 0.05, ns: not significant.

To investigate the function of BS-DRL1 in vivo, we generated BS-DRL1 knockout (KO) mice with CRISPR/Cas9 technology. BS-DRL1 KO mice exhibited normal gross brain anatomy and appeared to be healthy overall, with the exception of being a slightly smaller size (Supplementary Fig. 4a–d). For in vivo DDR evaluation, 3-month-old mice were subjected to gamma-irradiation (4 Gy), recovered for 3 h, and then sacrificed for immunostaining. We found that BS-DRL1 KO mice showed a significant increase in γH2AX immunostaining in NeuN-positive (NeuN+) cells in the cerebral cortical sections (Fig. 2a, b). Given that cerebellar dysfunction is observed in many neurological diseases caused by mutation of DDR genes^25^, we reasoned that BS-DRL1 deficiency may also affect the DDR in the cerebellum. To our surprise, instead of NeuN+ cells being the primary γH2AX immunoreactive cells in the cortex, NeuN-negative (NeuN−) cells adjacent to the granule cell layer were the primary γH2AX immunoreactive cells in the cerebellum (Fig. 2c, d). The number of NeuN+ cells with DNA damage in the cerebellum did not differ between BS-DRL1 KO and littermate controls (Fig. 2c, d). We further characterized the identity of these NeuN− cells with RNAscope and immunostaining, and found that in gamma-irradiated cerebellum sections, the cells that align outside of granular cell layer exhibit very higher BS-DRL1 RNAscope signal (Fig. 2e). Immunofluorescence co-staining further revealed that the majority of these cells are parvalbumin-positive (PV+) and calbindin-positive Purkinje cells, and the RNAscope signal of BS-DRL1 is mainly localized in the nuclear of these cells (Fig. 2f, g, and supplementary Fig. 4e–g). It is unclear whether the increased RNAscope signal of BS-DRL1 in Purkinje cells of brain sections is conferred by its concomitant increase of expression level, or the recruitment/relocation of BS-DRL1 to the DSB sites, as there is no increased expression of BS-DRL1 detected after the induction of DNA damage in primary culture neurons (Fig. 1d).

To examine whether BS-DRL1 KO mice exhibit higher endogenous DNA damage levels, we performed immunostaining with brain sections prepared from 3-, 6- and 12-month old KO and WT mice. No γH2AX difference was observed in NeuN+ cells in the cortex nor in the NeuN+ and NeuN− cells in the cerebellum of 3-month-old mice (Supplementary Fig. 5a–d,). For 6-month-old mice, there is a tendency, but not statistically significant increase of the number of NeuN− cells with more γH2AX foci (Supplementary Fig. 5e, f,). However, when we performed the same experiment with 12-month-old naive mice, a significant increase of γH2AX immunoreactivity was observed in PV+ neurons of BS-DRL1 KO cerebellum sections compared to controls (Fig. 2j, k). For NeuN+ cells in the cortex, there is a trend of increased γH2AX positive signal, but it is not statistically significant (Fig. 2h, i).

To further assess the consequences of the DDR impairment on the chromatin structure of the brain, we examined the immunoreactivity of heterochromatin protein 1 (HP1a), a key factor in genome stability and heterochromatin structure organization. We found that BS-DRL1 KO mice showed a significantly reduced HP1a immunoreactivity after gamma-irradiation (Supplementary Fig. 5g, h), indicating a more relaxed and disorganized chromatin structure and genome instability which may have been caused by the accumulation of unrepaired DNA breaks.

**Fig. 3:**
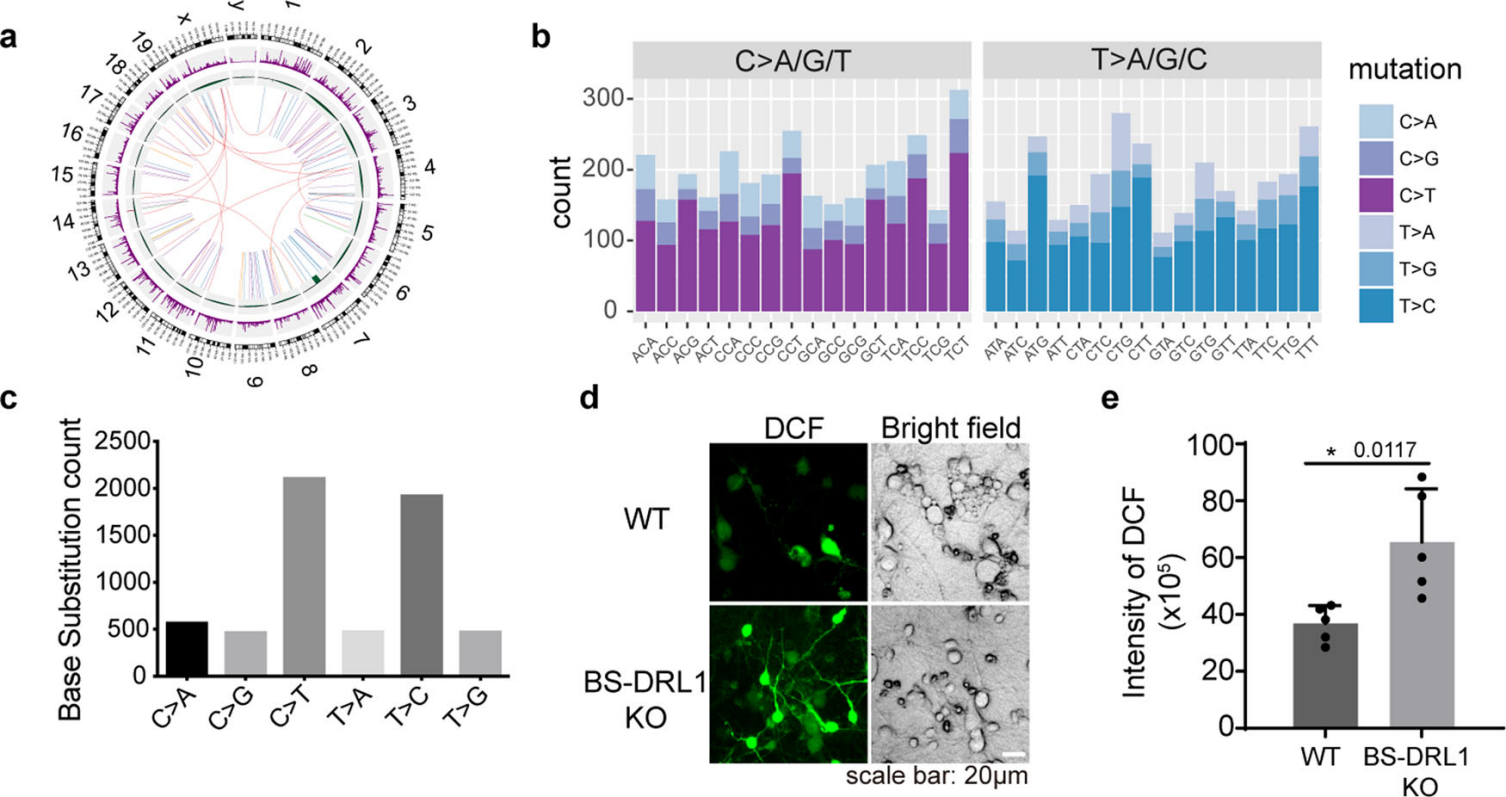
BS-DRL1 KO mice show genome instability. **a** Circos plot of 3-month-old mice from the whole-genome sequencing data of 3 pairs of BS-DRL1 KO mice brain tissues. The circle plot represents rearrangements (inner arcs), copy-number alternations (inner rings) and single nucleotide polymorphisms (middle rings). In rearrangements, lines show translocation breakend (red), deletions (blue), tandem duplication (green), insertions (purple), inversions (orange). Copy-number gain and loss regions are shown in red and green. **b**, **c** Summary of the base substitute in mutations identified by whole-genome sequencing of three pairs of BS-DRL1 KO mice brain tissues. **d**, **e** ROS level of primary neurons (DIV9) from BS-DRL1 KO mice and littermate controls were measured with an oxidation-sensitive fluorescent probe DCFH-DA (Dichloro-dihydro-fluorescein diacetate). Five images were used for statistics, Data are presented as mean ± SD, *p < 0.05.

To analyze the effect of DDR impairment on genome stability in naive BS-DRL1 KO mice, we performed whole-genome sequencing (WGS) for the brains of three pairs of 3-month-old BS-DRL1 KO mice and littermate controls. We identified 12,149 BS-DRL1 KO mice specific somatic mutations and indels, of which 8,241 (67.8%) were intergenic and intronic mutations, and 3039 (25%) were exonic mutations. A total of 21 frameshift indels and 18 of interchromosomal rearrangements were identified (Fig. 3a and Supplementary Fig. 6a, b). It is noteworthy to point out that there are almost no mutations/ indels were identified on Y chromosome in our WGS, indicating that Y chromosome is spared from DNA damage in BS-DRL1 null mice. We also performed RNA-Seq of brain tissues from three pairs of BS-DRL1 KO and littermate controls, and identified only a very small number of genes with significantly different expression levels, none of which are significantly related to brain function or DDR (Supplementary Fig. 6c).

In addition, we noticed that there was a DNA fragment depletion localized to the intron region of the gamma-aminobutyric acid type A receptor gamma3 (*Gabrg3)*, downstream of BS-DRL1. It is unclear whether this depletion originated from the functional deficiency of BS-DRL1 or from a mistargeting of CRISPR/Cas9 system. To rule out the possibility of off-target effects of CRISPR/Cas9, we validated that the expression of *Gabrg3* and 13 other members of the GABA receptor family were unchanged between BS-DRL1 KO and littermate controls (Supplementary Fig. 6d).

To gain insight into the most relevant DNA damage type related to the loss-of-function of BS-DRL1 in vivo, we analyzed the base substitution spectrum of all mutations identified in BS-DRL1 KO brains and observed that dominant C>T and T>C base transition rates account for ~75% of all mutations (Fig. 3b, c). Previous studies have suggested that the high proportion of T-to-C and C-to-T transition mutations was positively correlated with reactive oxygen species (ROS)^26^. As such, we set out to check the levels of ROS in BS-DRL1 KO neurons. Indeed, ROS levels were markedly higher in BS-DRL1 KO neurons than those of WT controls (Fig. 3d, e), indicating that the loss-of-function of BS-DRL1 leads to an increased level of ROS in physiological conditions in vivo, which may ultimately lead to the accumulation of oxidative DNA damage.

### BS-DRL1 interacts with HMGB1 to regulate DDR

**Fig. 4:**
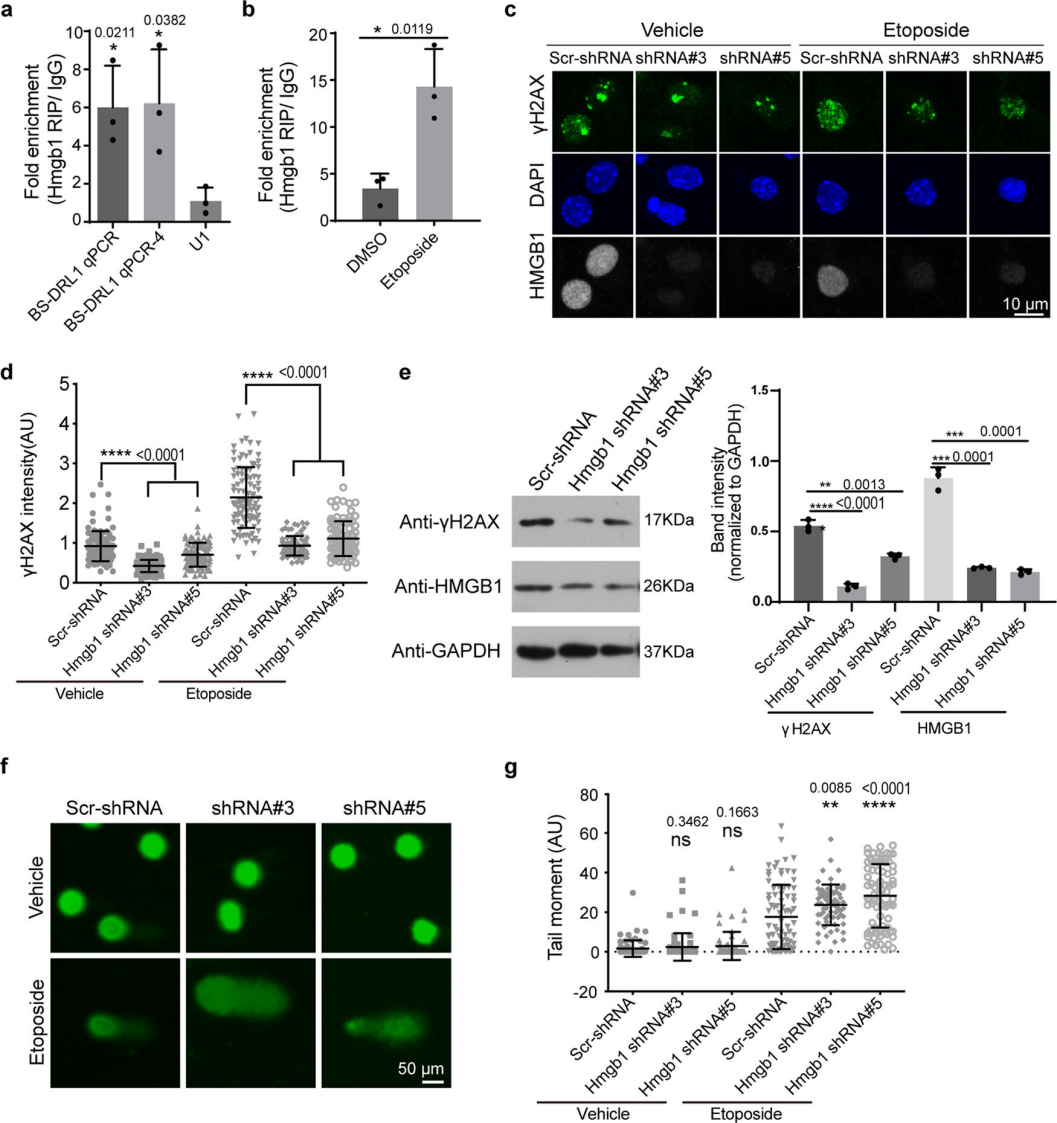
BS-DRL1 functions by interacting with HMGB1. **a** Interaction of HMGB1 and BS-DRL1 in primary neurons was confirmed by RNA immunoprecipitation (RIP) with HMGB1 antibody following by RT-qPCR, *U1* RNA was used as a negative control. Data are presented as mean ± SD. *n* = 3. **b** RIP-(RT-qPCR) showed an increased interaction of HMGB1 and BS-DRL1 in neurons upon DNA damage. Primary neurons were treated with DMSO or ETO for 1 h and harvested for following experiments. Data are presented as mean ± SD. *n* = 3. **c**, **d** DNA damage in HMGB1 KD neurons was evaluated by γH2AX immunostaining. Primary neurons infected with indicated shRNAs virus were treated with vehicle or etoposide for 1 h and stained with γH2AX and HMGB1 antibodies. Scale bar: 10 μm. Data are presented as mean ± SD. *n* = 85 neurons. ****p < 0.0001. AU, arbitrary units. **e** The level of γH2AX in neurons expressing HMGB1-shRNA was measured by western blot analysis. Primary neurons infected with indicated shRNAs virus were treated with vehicle or ETO for 1 h before harvesting for Western blotting with indicated antibodies. Data are presented as mean ± SD. **p < 0.01, ***p < 0.001, ****p < 0.0001. *n* = 3 biologically independent experiments. **f**, **g** Comet assay to measure the DNA damage accumulation in neurons. Primary neurons infected with indicated shRNA virus were treated with vehicle or ETO for 1 h and then proceeded for comet assay. Scale bar: 50 μm. Data are presented as mean ± SD. *n* = 63 neurons. ****p < 0.0001, **p < 0.01, ns not significant, AU arbitrary units.

It is critical to identify the interacting proteins with a given lncRNA to understand the molecular mechanism regarding its function, so we conducted CHIRP-MS (Comprehensive Identification of RNA-binding proteins by mass spectrometry) to identify the proteins interacting with BS-DRL1^27^. Our CHIRP-MS analysis revealed that HMGB1, a vital chromatin-associated non-histone protein, binds BS-DRL1 (Supplementary Fig. 7a, b). This result was confirmed with RNA immunoprecipitation followed by qPCR (RIP-qPCR, Fig. 4a). Furthermore, the interaction between BS-DRL1 and HMGB1 is mainly mediated by the N-terminus of the HMGB1, and the chromatin binding proportion of HMGB1 is increased after the induction of DNA damage (Fig. 4b, Supplementary Fig. 7c, d), whereas the expression of HMGB1 was unchanged in ETO treated neurons or BS-DRL1 KO brain tissue (Supplementary Fig. 7e, f), suggesting that BS-DRL1 and HMGB1 form a complex in response to DNA damage.

HMGB1 plays a critical role in replication, chromatin remodeling, DNA repair, and genome stability^16^. To assess the function of HMGB1 in neuronal DDR, primary cultured neurons expressing Scr-shRNA or HMGB1-shRNA were treated with ETO and stained for γH2AX immunoreactivity. We observed that while neurons expressing Scr-shRNA exhibited robust γH2AX staining in response to ETO treatment, neurons expressing HMGB1-shRNA showed a diminished γH2AX signal upon ETO treatment (Fig. 4c, d). Consistent with immunostaining, western blot analysis also detected a decreased γH2AX band in neurons expressing HMGB1-shRNA compared to Scr-shRNA infected neurons (Fig. 4e, please see Supplementary Fig. 7g for the full length of gel). Notably, the result of decreased γH2AX with HMGB1-shRNA is the opposite with what we observed in BS-DRL1 KD neurons in which γH2AX is increased (Fig. 1e–h).

To directly assess the actual level of DNA damage following HMGB1 KD, we performed a comet assay, as was done previously for BS-DRL1. Vehicle-treated neurons with or without HMGB1 KD both showed few comet tails, indicating no or few DNA strand breaks. In contrast, ETO-treated neurons expressing HMGB1*-*shRNA showed markedly increased tail moments compared to cells expressing Scr-shRNA, indicating an increase of DNA breaks (Fig. 4f, g). Thus, despite the accumulation of more DNA strand breaks, HMGB1 KD resulted in reduced DDR signaling, as evidenced by diminished γH2AX both in immunostaining and western blot analyses.

**Fig. 5:**
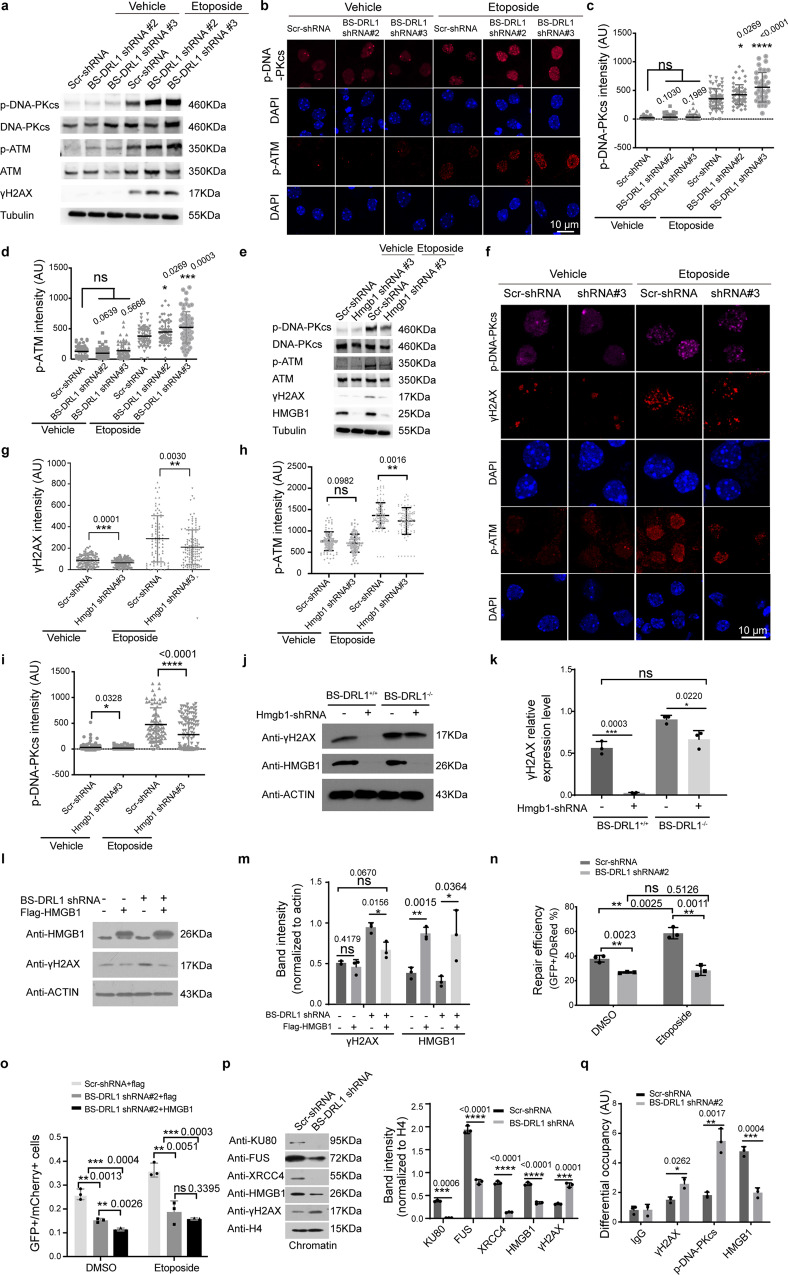
DNA damage response (DDR) mediated by BS-DRL1 and HMGB1. **a** Enhanced DDR of BS-DRL1 KD neurons demonstrated by western blot analysis. Primary cortical neurons were transfected with BS-DRL1 shRNA or Scr-shRNA virus, treated with vehicle or ETO for 1 h and followed by western blot with antibodies against ATM, DNA-PKcs, p-ATM (S1981), p-DNA-PKcs (S2056), and γH2AX. Tublin was used as an internal control. **b**–**d** Enhanced DDR of BS-DRL1 KD neurons demonstrated by immunofluorescence staining. Primary cortical neurons infected with indicated shRNA virus, treated with vehicle or ETO for 1 h and stained with antibodies against p-ATM and p-DNA-PKcs. Scale bar: 10 μm. The fluorescence intensity of p-ATM and p-DNA-PKcs signal in the GFP positive cells was measured and quantified (the shRNA has GFP tag). Data are presented as mean ± SD. *n* = 40 neurons. *p < 0.05, ****p < 0.0001, ns not significant, AU arbitrary units. **e** Impaired DDR of HMGB1 KD neurons demonstrated by western blot analysis with antibodies against ATM, DNA-PKcs, p-ATM, p-DNA-PKcs, HMGB1, and γH2AX. Primary cortical neurons transfected with HMGB1 shRNA or Scr-shRNA virus were treated with vehicle or ETO for 1 h and harvested for western blot. **f**–**i** Impaired DDR of HMGB1 KD neurons demonstrated by immunofluorescence staining. Primary cortical neurons infected with indicated shRNA virus, treated with vehicle or ETO for 1 h and immunolabeled with indicated antibodies (γH2AX, p-ATM, and p-DNA-PKcs). Scale bar: 10 μm. The quantification showed the fluorescence intensity of γH2AX, p-ATM, and p-DNA-PKcs. Data are presented as mean ± SD. *n* = 40 neurons. *p < 0.05, **p < 0.01, ***p < 0.001, ****p < 0.0001, ns not significant, AU arbitrary units. **j**–**k** Western blot analysis of γH2AX in BS-DRL1 KD, HMGB1 KD, or BS-DRL1/HMGB1 double deficiency neurons. Primary cortical neurons from WT or BS-DRL1 KO mice were transfected with HMGB1 shRNA or Scr-shRNA virus and treated with ETO for 1 h and followed by western blot with indicated antibodies. Data are presented as mean ± SD, *n* = 3. *p < 0.05 ****p < 0.0001. **l**, **m** Primary cortical neurons transduced with either BS-DRL1-shRNA, flag tagged HMGB1 overexpression virus or both were treated with ETO for 1 h and processed for western blot with indicated antibodies. Data are presented as mean ± SD, *n* = 3. *p < 0.05, **p < 0.01, ****p < 0.0001, ns not significant. **n** Reduced NHEJ repair in BS-DRL1 KD neurons. Primary cortical neurons transduced with either BS-DRL1-shRNA or scr-shRNA were subjected to NHEJ reporter assay. Data are presented as mean ± SD. **p < 0.01, ns not significant. *n* = 3 biologically independent experiments. **o** Primary cortical neurons transduced with either BS-DRL1-shRNA, flag or flag tagged HMGB1 overexpression virus and DSB repair efficiency was evaluated as in (**p**). Data are presented as mean ± SD. **p < 0.01, ****p < 0.0001, ns not significant. *n* = 3 biologically independent experiments. **p** The assembly of HMGB1 and other DDR proteins on chromatin was examined by chromatin fractionation and western blot analysis. Primary cortical neurons transduced with BS-DRL1 shRNA or Scr-shRNA virus were treated with ETO for 1 h and processed for subcellular fractionation and western blot analysis. Data are presented as mean ± SD. ***p < 0.001, ****p < 0.0001. *n* = 3 biologically independent experiments. **q** The occupancy of γH2AX, p-DNA-PKcs, and HMGB1 at DSBs sites was assessed using CHIP assays in primary neurons transduced with BS-DRL1 shRNA or Scr-shRNA following I-Ppo-I introduction. Data are presented as mean ± SD. *p < 0.05, ***p < 0.001, ****p < 0.0001. *n* = 3 biologically independent samples.

To gain insight into the opposite effects of the BS-DRL1 KD and HMGB1 KD on γH2AX, we examined whether BS-DRL1 and HMGB1 affect the DNA damage regulatory molecules upstream of γH2AX in DDR signaling cascade. We focused our study on ATM and DNA-PKcs, two kinases that are known to phosphorylate H2AX upon DNA damage, and have been widely regarded as major mediators for DSB recognition to initiate the DDR. We found that although the baseline level of total ATM and DNA-PKcs were seems slightly increased, the induction of DNA damage by ETO treatment markedly enhanced the γH2AX, p-ATM, and p-DNA-PKcs in BS-DRL1 KD neurons, depicted by both western blot and immunostaining (Fig. 5a–d, Supplementary Fig. 7h). In contrast, a decrease of p-ATM and p-DNA-PKcs were detected in ETO treated HMGB1 KD neurons, which is consistent with the reduction of γH2AX demonstrated in the same conditions (Fig. 5e–i, Supplementary Fig. 7i), indicating that HMGB1 is essential for the initial sensing and signaling of DNA damage.

The opposite effect of BS-DRL1 and HMGB1 on the phosphorylation of ATM, DNA-PKcs and subsequently H2AX, and their enhanced interaction upon DNA damage, prompted us to hypothesize that BS-DRL1 may suppress the phosphorylation of ATM and DNA-PKcs to prevent the erroneous or over activation of DDR to protect the integrity of genome and prevent neurons from death; whereas the HMGB1 may function as a DNA damage sensor to promote phosphorylation of ATM, DNA-PKcs to initiate the DDR signaling cascade in response to DNA damage and other genotoxic insult, and the interaction between BS-DRL1 and HMGB1 is essential for the HMGB1 to execute its function in DDR and DNA repair.

Based on this hypothesis, one would expect that in the wild type (WT) neurons expressing normal level of BS-DRL1, knockdown of HMGB1 will lead to a decrease of γH2AX, while knockdown of BS-DRL1 will render the cells more susceptible to DNA damage and concomitantly increase the level of γH2AX. Indeed, these are actually what we observed as aforementioned (Fig. 1e, f, Fig. 4c–e). To gain further insight into their cooperative interaction in neuronal DDR, we continued to assess the γH2AX level in BS-DRL1 and HMGB1 double deficient neurons. We found that the γH2AX level of ETO-treated HMGB1KD/BS-DRL1 KO neurons is comparable to the ETO-treated WT neurons, but lower than the BS-DRL1 KO neurons expressing intact HMGB1 (Fig. 5j, k), and higher than the ETO treated HMGB1 KD neurons expressing intact BS-DRL1. Considering that under all these three conditions, i.e., BS-DRL1 KO, HMGB1 KD, and BS-DRL1 KO/HMGB1 KD, the neurons actually accumulated more DNA damage (Fig. 1h, i; Fig. 4f, g, Supplementary Fig. 7j), these results collectively suggest that HMGB1 is an apical component for the neurons to sense and signal the DDR, and an indispensable effector for BS-DRL1 mediated DDR.

We next set up to examine the effect of HMGB1 overexpression (OE) on γH2AX in BS-DRL1 KD neurons to further define their interplay and regulatory role in DDR. We infected WT and BS-DRL1 KD neurons with the HMGB1-Flag OE virus and then detected the γH2AX levels with western blot. Our results revealed that the OE of HMGB1 has no, or a very little effect on the γH2AX in the ETO-treated WT neurons, which can be reasonably explained by the presence of normal expression of BS-DRL1; however, to our surprise, OE of HMGB1 restored the upregulation of γH2AX in BS-DRL1 KD neurons to a level comparable to the WT neurons after the induction of DNA damage (Fig. 5 l, m), but the amount of accumulated DNA damage was remained upregulated (Supplementary Fig. 7k). Taken together, these results suggest that the reduction of γH2AX by OE of HMGB1 in BS-DRL1 KD neurons is not because the improvement of DNA repair. As such, for neurons with the disrupted expression of BS-DRL1, despite the level of γH2AX and HMGB1, the DNA repair efficiency is compromised, indicting a very important role of BS-DRL1 in the neuronal DNA repair.

The slower recover of γH2AX level showed in Fig. 1g already suggests an impairment of DNA repair in neurons infected with the BS-DRL1 shRNAs. To further corroborate this result, we continued to assess the DNA repair efficiency of neurons using a previously described NHEJ reporter assay^12^. We found that, compared with cells infected with Scr-shRNAs, knockdown of BS-DRL1 dramatically reduced the NHEJ-mediated DSB repair (Fig. 5n). We then determined whether the HMGB1 is sufficient for mediating the function of BS-DRL1 in DNA repair with a rescue experiment. We overexpressed HMGB1 in neurons infected with either Scr-shRNA or BS-DRL1-shRNA, and assessed the DNA repair efficiency with HNEJ reporter assay again. We found that OE of HMGB1 did not rescue the DNA repair deficiency in neurons infected with BS-DRL1 shRNA virus (Fig. 5o), nor the accumulation of DNA strand breaks (Supplementary Fig. 7k), indicating that supplement HMGB1 alone is not sufficient to compensate the DNA repair deficit caused by loss function of BS-DRL1, there must have other critical DNA repair related proteins also involved in the DS-DRL1 mediated DNA repair. Together, these results suggest that BS-DRL1 is important for NHEJ mediated DSB repair, and HMGB1 is one of the components of the DNA repair complex that mediate the function of BS-DRL1 in DNA repair.

Proteins involved in chromatin modification or DNA repair dynamically interact with chromatin to regulate accessibility of DNA, to control gene transcription and DNA repair, and to maintain genome integrity. The extent of their association with chromatin changes rapidly in response to genomic insults. To determine whether BS-DRL1 is necessary for the assembly of DNA repair complexes, particularly HMGB1, to the damaged DNA foci, we examined the binding of HMGB1 on chromatin in the neurons following ETO treatment. We extracted cellular chromatin fractions from neurons expressing either Scr-shRNA or BS-DRL1-shRNA following ETO treatment and measured the amount of HMGB1 binding to the chromatin. We observed that the amount of chromatin-associated HMGB1 was significantly reduced in BS-DRL1 KD neurons compared to controls (Fig. 5p). We simultaneously checked the association of chromatin with two other critical DDR proteins, Ku80 and XRCC4, both of which are required for non-homologous end joining (NHEJ) DNA repair. We found that these two proteins also displayed reduced chromatin binding in ETO-treated BS-DRL1 KD neurons (Fig. 5p). The reduction of the chromatin-bound HMGB1, Ku80, and XRCC4 upon BS-DRL1 KD was not due to changes in their expression, as revealed by the western blot analysis of whole-cell lysate (Supplementary Fig. 7l). Furthermore, we found that depletion of BS-DRL1 also affected the interaction of HMGB1 with LIG4, a DNA ligase that is essential for DSB repair through NHEJ (Supplementary Fig. 7m).

We previously showed that FUS, an ALS risk gene, plays a pivotal role in DNA damage response and repair^12^. Interestingly, we found that the chromatin binding of FUS in response to DNA damage was also decreased in BS-DRL1 KD neurons (Fig. 5p). We used HITS-CLIP to confirm the interaction of FUS with BS-DRL1 and found that there is a trend toward an increase of FUS/BS-DRL1 interaction upon DNA damage, although this was not statistically significant (Supplementary Fig. 7n). The biological importance of this observation merits further study in the future in the context of disease.

We further conducted an I-PpoI based assay to assess the recruitment of HMGB1 to the DNA damage sites. I-PpoI is a rare-cutting homing endonuclease that can generate DSBs at defined genome loci, and thus has been used for precisely assess the enrichment of proteins approximating to the DSBs^28,29^. I-PpoI induction followed by Chromatin immunoprecipitation (ChIP) and qPCR (ChIP-qPCR) revealed that the occupancy of HMGB1 is dramatically decreased at I-PpoI-generated DSBs in BS-DRL1 KD neurons (Fig. 5q). Interestingly, the enrichment of p-DNA-PKcs and γH2AX at I-PpoI-generated DSBs is increased, which is consistent with our western blot and immunostaining analysis (Fig. 1e, f; Fig. 5a–d).

Taken together, these results suggest that BS-DRL1 is important for the assembly and stable retention of HMGB1 and other critical DNA repair complexes on DNA damage foci in neurons, and even though the initial events of DNA damage signaling are properly activated, BS-DRL1 KD neurons exhibits a deficit in DNA repair and consequently, an accumulation of more damaged DNA.

### BS-DRL1 KO mice show impaired motor function and progressive Purkinje cell degeneration

**Fig. 6:**
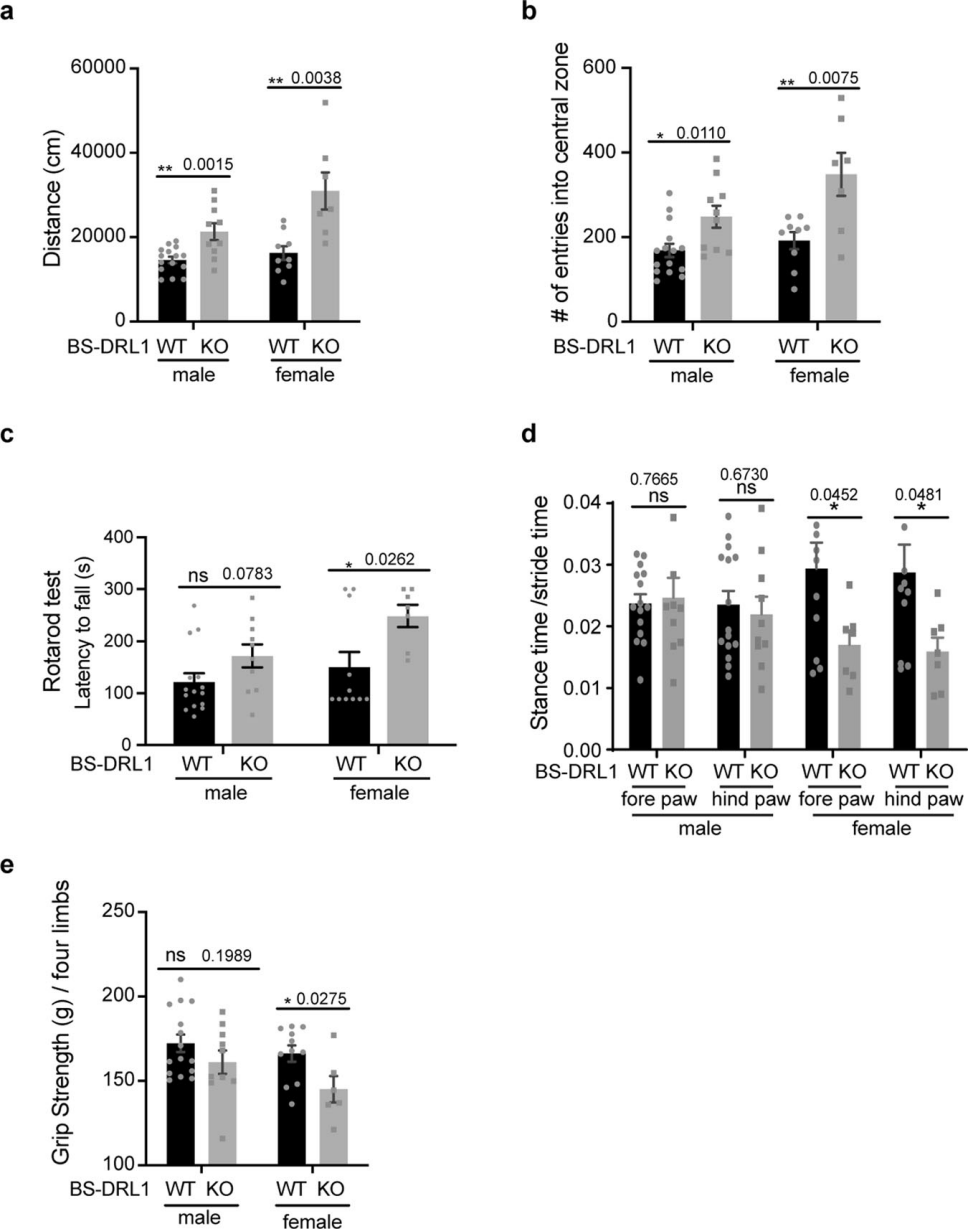
BS-DRL1 KO mice show motor deficiency. **a**, **b** Open field test showing significantly higher total traveling distance and more times of entry into the central zone of the arena in 6-month-old BS-DRL-1 KO mice compared to the littermate controls. Data are presented as mean ± SD. Male, *n* = 15:10; female, *n* = 9:7. *p < 0.05, **p < 0.01. **c** An increase of latency to fall was observed in rotarod test of 6-month-old BS-DRL-1 KO female mice compared to the littermate controls. Data are presented as mean ± SD. Male, *n* = 15:10; female, *n* = 11:7. *p < 0.05, ns not significant. **d** Gait analysis of 6-month-old BS-DRL-1 KO female mice. Representative example shows the percentage of stance time/stride time of four limbs. Data are presented as mean ± SD. Male, *n* = 15:10; female, *n* = 11:7. *p < 0.05, ns not significant. **e** Grip strength is impaired in BS-DRL-1 KO female mice compared to the littermate controls. Data are presented as mean ± SD. Male, *n* = 15:10; female, *n* = 11:7. *p < 0.05.

The in vitro and in vivo data suggested that BS-DRL1 plays a critical role in the DDR in neurons. To further assess the importance of this physiological function in vivo, we examined the consequence of BS-DRL1 depletion at the behavioral level. We performed a number of behavioral tests to evaluate the phenotypes in the 6-month-old BS-DRL1 KO mice. The open field test has been used widely in rodents to evaluate the general activity and exploratory behavior. We observed that for BS-DRL1 KO mice, total traveling distance and times of entry into the central zone of the arena were significantly higher than that of the littermate controls, suggesting that BS-DRL1 mice are more active (Fig. 6a, b). In an accelerating rotarod test, an increased latency to fall was only observed in female BS-DRL1 KO mice. There was an increasing trend in male mice as well, but this was not statistically significant (Fig. 6c). There were no differences between BS-DRL1 KO mice and littermate controls in the pole test (Supplementary Fig. 8a).

Gait analysis is widely used to evaluate motor functions, such as balance and coordination. We used treadmill-based gait analysis to assess several aspects of gait behavior, including stride, stance, and pressure. We observed a significant decrease of the stance time and stride frequency in both forelimbs and hindlimbs, as well as a dramatic decrease in the mean pressure intensity of the right forelimb, left forelimb, and right hindlimb in female BS-DRL1 KO mice (Fig. 6d and Supplementary Fig. 8b). The grip strength of the four limbs was also decreased in female KO mice (Fig. 6e). Interestingly, no significant change was observed in male mice in the gait analysis and grip strength test.

**Fig. 7:**
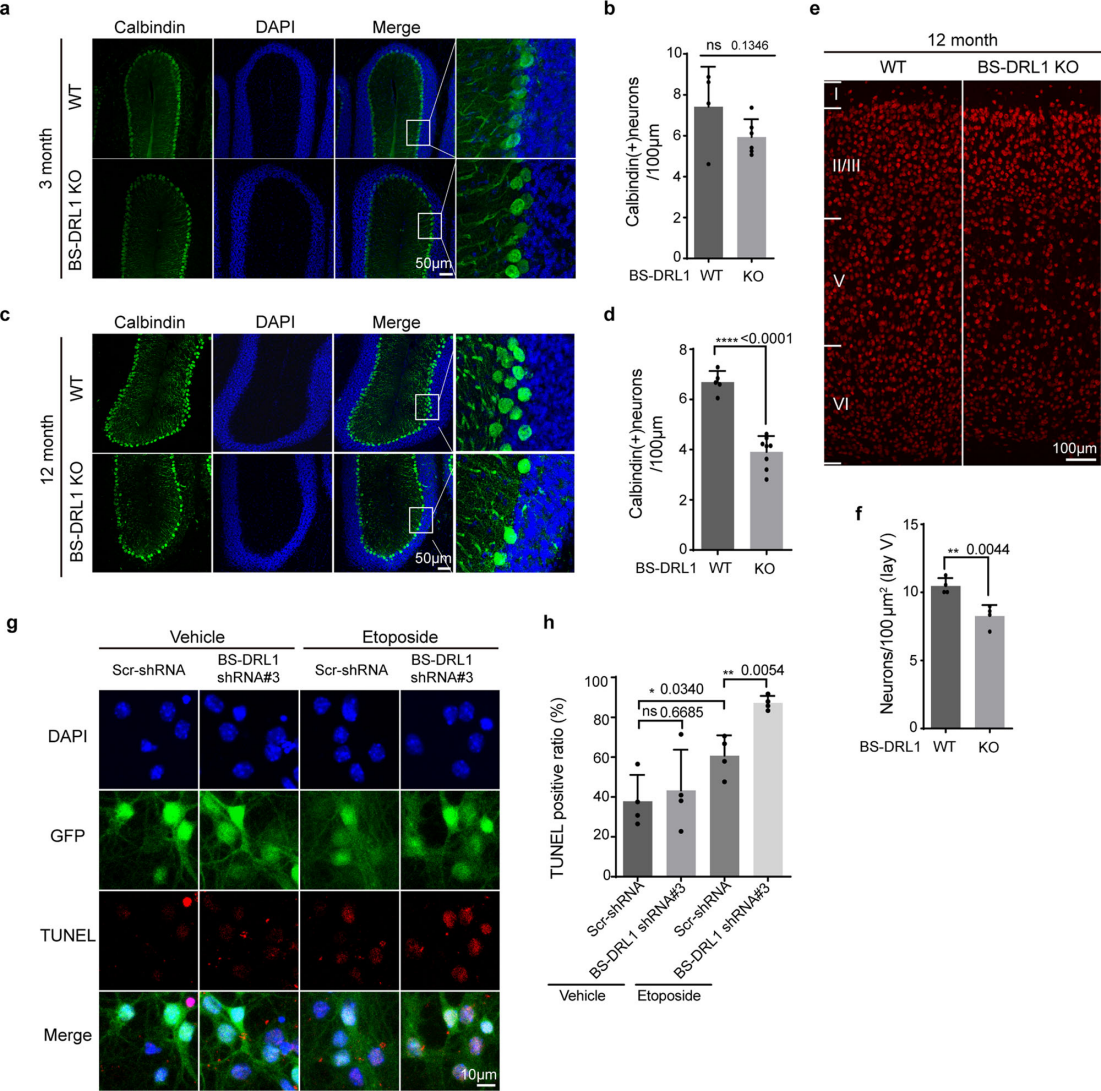
BS-DRL1 KO mice show progressive Purkinje cell degeneration. **a**, **b** Representative images showing immunostaining of Purkinje cells (in green) and quantification of Purkinje cells. Immunofluorescence staining was performed with brain sections prepared from 3-month-old naive mice and calbindin antibody. Scale bar: 50 μm. Data are presented as mean ± SD. For each group, *n* = 3. ns not significant. **c**, **d** Same as (**a**, **b**) except the brain sections were prepared from 12-month-old naive mice. Scale bar: 50 μm. Data are presented as mean ± SD. For each group, *n* = 3. ****p < 0.0001. **e**, **f** Representative images of immunostaining and quantification for NeuN-positive neurons in M1 cortex of naive 12-month-old mice. Scale bar:100 μm. Data are presented as mean ± SD. For each group, *n* = 3. **p < 0.01. **g**, **h** TUNEL staining and quantification of neurons transduced with indicated shRNA virus and treated with vehicle or ETO for 1 h. Four images per group and 28–34 cells per image were used for statistic. Scale bar:10 μm. Data are presented as mean ± SD. *p < 0.05, **p < 0.01, ns not significant. *n* = 3 biologically independent samples.

Motor functions, such as coordination and balance, are mainly controlled by the cerebellum, and the Purkinje cell is a significantly affected cell type in a number of DDR-related gene KO mouse models^30^. We performed immunostaining with cerebellar sections using anti-calbindin antibody to examine whether the manifestation of gait impairment might be associated with the loss of Purkinje cells in the cerebellum of BS-DRL1 KO mice. We found that while there is a dramatic decline in the number of Purkinje cells in 12-month-old BS-DRL1 KO mice, only a slight decline in 6-month-old but not in 3-month-old mice were detected when compared to age-matched controls (Fig. 7a–d, Supplementary Fig. 9a–c), indicating an age-dependent progressive loss of Purkinje cells.

We then performed a Golgi staining to further analyze the morphology of Purkinje cells using *Sholl* analysis with 12-month-old mice brain tissues, and found that there are no significant changes of the complexity of dendrites between WT and KO mice (Supplementary Fig 10a, b). Moreover, we also detected a slightly reduced number of NeuN+ cells in layer 5 of the motor cortex in 12-month-old BS-DRL1 KO mice (Fig. 7e, f). Interestingly, a survey for the expression level of the HMGB1 in 12-month-old BS-DRL1 KO mice revealed that HMGB1 is also highly enriched in Purkinje cells of cerebellum, however, its expression level is not altered in BS-DRL1 KO condition (Supplementary Fig. 10c).

To examine whether the loss of the Purkinje cells in BS-DRL1 KO mice might be the consequence of impaired DDR, we treated primary cultured neurons infected with Scr-shRNA or BS-DRL1-shRNA virus with ETO and performed a TUNEL assay, which is widely used to identify and quantify apoptotic cells. We found that ETO treatment induced ~80% TUNEL positive cells in BS-DRL1 KD neurons compared to ~50% TUNEL positive cells in Scr-shRNA expression neurons. There was no significant difference between Scr-RNA and BS-DRL1-shRNA expressing neurons treated with vehicle (Fig. 7g, h). In support of this data, we also detected an increase in the number of cells with more p-ATM immunoreactivity in the 6-month-old BS-DRL1 KO mice with a concomitant, though mild degeneration of the Purkinje cells (Supplementary Fig. 9d).

## Discussion

Here, we report the characterization of a lncRNA with enriched expression in the brain that we termed BS-DRL1 based on its expression and function. We found that BS-DRL1 is important for mediating the DDR in neurons both in vitro and in vivo, and that BS-DRL1-mediated DDR exhibits brain region and cell-type specificity. Behavioral tests demonstrated that BS-DRL1 depletion in mice leads to impaired locomotion and motor coordination that was concomitant with increased neuronal degeneration.

### BS-DRL1 is a brain-specific lncRNA involved in DNA damage response

Our study of this lncRNA has yielded several important and unexpected findings. First, we identified multiple new BS-DRL1 transcripts that were previously not annotated and are brain-specific. Our studies suggest that for largely unexplored lncRNAs, despite extensive bioinformatic annotations and computational in silico predictions, there is a need to ultimately test and interrogate the *bona fide* transcripts experimentally, especially those with tissue-specific expression patterns. These experiments should include rapid amplification of cDNA ends (RACE) or related methods to define the 5′ and 3′ ends of the transcripts, coupled with RT-PCR or re-analysis of high-throughput sequencing data generated by relevant cell types or tissues.

Secondly, although lncRNAs display more tissue-specific expression patterns than protein-coding genes, particularly in the brain^24^, only very few brain-specific lncRNAs have been functionally characterized to date^31^. We show that BS-DRL1 is highly conserved between mouse and human (the exon region has about 85% identity, Supplementary Fig. 1b), specifically expressed in the brain and plays a pivotal role in DDR and DNA repair, expanding our understanding of lncRNA functions in neurons. The underlying mechanism for the brain-specific expression of BS-DRL1 is currently unclear; however, bioinformatic analyses show multiple REST binding sites in its promoter region (ENCODE3). Considering that REST has been well-documented as a master regulator of neuronal genes, it is not surprising that BS-DRL1 is expressed specifically in the brain.

Recent studies have highlighted the importance of genome stability in brain aging and neurodegeneration^8^. Increased DNA damage is one of the hallmarks of aging and is a key mechanism that contributes to the gain- and loss-of-function of neuronal gene expression and impaired brain function^9^. Therefore, the mechanisms that give rise to genomic instability have been the subject of intensive research in recent years. Our finding establishes that BS-DRL1 is an important regulator of the DDR in neurons and that there is an increased accumulation of DNA damage in 12-month-old, but not 3- and 6-month-old, BS-DRL1 KO mice brain (Fig. 2), suggesting that BS-DRL1 is necessary for the maintenance of genome stability during normal brain aging. It remains undetermined whether dysregulation of BS-DRL1 contributes to genome instability in neurodegeneration. Although direct involvement of BS-DRL1 in human disease has not been reported yet, our data demonstrate that BS-DRL1 interacts with the ALS disease gene FUS, and their interaction is likely increased upon DNA damage; this suggests that BS-DRL1 may be, at least indirectly, involved in the pathogenesis of FUS-ALS. Nevertheless, in light of our findings, a more thorough examination of this pathway in brain aging and neurodegeneration is warranted.

### BS-DRL1 regulates DDR by interacting with HMGB1

A common emerging theme of lncRNAs is that they typically form RNA-protein complexes to carry out their functions. LncRNAs can act as decoys, scaffolds, or guides to recruit proteins such as transcription factors and chromatin modifiers into a functional complex or specific genomic locus to regulate gene expression, chromatin structure, DDR, cell cycle, etc. We found that BS-DRL1 interacts with HMGB1, and this interaction is important for the assembly of the DDR complex and for maintaining genomic stability. These observations are consistent with previous studies that showed HMGB1 functions in DNA repair and genome stability^19,32^.

HMGB1 is a highly abundant protein with roles in several cellular processes, including chromatin structure and transcriptional regulation, as well as an extracellular role in inflammation^15,16^. It has been shown previously that overexpression of HMGB1 can attenuate the upregulated γH2AX level and protect Purkinje cell from death in a mutant Ataxin-1 knock-in (Atxn1-KI) mice model^18^, suggesting that HMGB1 is very important for survival of Purkinje cells. In line with this study, we found that BS-DRL1 and HMGB1 interact and work coordinately to regulate the DDR of neuronal cells, particularly Purkinje cells, and BS-DRL1 is essential for the survival of Purkinje cells during normal aging. Mechanistically, we propose that the functions of BS-DRL1 in DDR and DNA repair are twofold: (1) be both opposite and complementary with HMGB1 to regulate the initial events of DDR, such as phosphorylation of ATM, DNA-PKcs and consequently, their target H2AX, to enable the neurons to precisely cope with the DNA damage; (2) to facilitate the assembly and retention of repair complexes to the DNA damage foci, which including but  are not limited to HMGB1 and other critical DNA repair proteins such as XRCC4 and LIG4 to promote DNA repair (please see Supplementary Fig. 11 for a working model). BS-DRL1 is likely essential for the recruitment and retention of HMGB1 containing proteins complex on the chromatin. It is possible that the coordinated dysregulation of BS-DRL1 and HMGB1 interactions would produce a state of severe genomic instability, as evidenced by increased accumulation of DNA damage, reduced assembly of HMGB1 on chromatin and decreased HP1a immunoreactivity upon BS-DRL1 depletion. Similarly, a number of lncRNAs have been shown to couple with its interacting proteins to regulate chromatin conformation changes in response to DNA damage. For instance, the expression of lncRNA NORAD is upregulated in response to DNA damage and hypoxia^6,33^. NORAD KO cells exhibit chromosomal instability and aneuploidy by down-regulating its target PUMILIO, pum1 (Pumilio homolog 1) haploinsufficiency in mice causes neurodegeneration^6,34^.

We also note that the interaction of BS-DRL1 and HMGB1 was identified and confirmed by CHIRP-MS and RIP-qPCR, respectively. However, we were unable to identified BS-DRL1 as a HMGB1 interacting RNA with high-throughput sequencing of RNA isolated by crosslinking immunoprecipitation (HIT-CLIP), which is widely used for mapping the protein-RNA interactions^35^, indicating that HMGB1 might indirectly interact with BS-DRL1. Further research is needed to determine the proteins directly interacting with BS-DRL1 in neurons. In this regard, we identified FUS/BS-DRL1 interaction with HITS-CLIP using antibody against FUS (but failed to capture FUS with CHIRP-MS assay), but depletion of FUS does not affect the interaction between HMGB1and BS-DRL1, suggesting that FUS is not involved in meditating the interaction of BS-DRL1 and HMGB1.

### BS-DRL1 KO mice exhibit impaired DDR, motor dysfunction, and neurodegeneration

We find that the knockout of BS-DRL1 results in DDR impairment, motor deficits, and neuronal loss. Interestingly, the DDR mediated by BS-DRL1 exhibits brain sub-region and cell-type specificity. In the cortex, the major response cells are NeuN+ neurons, while in the cerebellum, the major response cells are NeuN−, PV+ cells. Further analysis revealed that these cells are, at least partly, Purkinje cells (Figs. 2 and  7). Given that BS-DRL1 is enriched in the brain, these data suggest that BS-DRL1 is of particular importance and is a key factor for mediating DDR in neurons, and its expression and function may be modulated in different brain regions or neuronal subtypes in order to titrate distinct DDR and repair mechanisms.

In support of this notion, increasing evidence has revealed that patients or animal models with mutations in key DNA repair genes often exhibit tissue-specific phenotypes^36^. For example, age-related motor neuron degeneration has been observed in mice lacking ERCC1^37^, indicating that the accumulation of DNA damage due to the impairment of nucleotide excision repair pathway contributes to the motor neuron vulnerability. ATM deficiency is also linked to increased oxidative stress within the cerebellum, the brain region heavily affected in Ataxia Telangiectasia patients^38^. Moreover, mutations in proteins involved in single-strand break repair tend to lead to impaired neurological functions, as is the case for ataxia^8^. Taken together, these studies support the view that DNA damage is modulated differentially between tissues. However, we know surprisingly little about how the DDR in different brain regions and cell-types is regulated, due in part to the lack of cellular and animal models. We suggest that BS-DRL1 mice is a good model for studying the mechanism underlying brain- and cell-specificity of DDR.

Since each lncRNA has multiple protein binding targets, the cell-type specific DDR of BS-DRL1 may be influenced by the availability and specificity of its interacting proteins, such as HMGB1 described in this study, under different conditions. In line with this view, the expression level of HMGB1 is dramatically decreased in the degenerating motor neurons, but was remarkably increased in the reactive glia cells in ALS mouse model and human patients^39^, suggesting that the expression of HMGB1 may be regulated in different types of cells and conditions, which may lead to a cascade of different events that underlie cell-type specific vulnerability.

Notably, the locomotion deficiency is dominantly presented in female BS-DRL1 KO mice, so does Purkinje cell degeneration and accumulation of DNA damage. Coincidently, there were almost no mutations and indels were identified on Y chromosome of BS-DRL1 KO mice, indicating that Y chromosome is spared from damage. It is currently unclear whether this gender difference was associated with some uncovered function of BS-DRL1 on the differential regulation of the genome integrity of sex chromosome, or the expression of some male-specific genes/non-coding RNAs compensated the disrupted functions of BS-DRL1, or protected the genome of BS-DRL1 KO mice. Nevertheless, this is an interesting question that merits further investigation.

It is of particular interest that the major mutations induced by depletion of BS-DRL1 are T–C and C–T substitutions. This observation supports the relevance of BS-DRL1 in oxidative DNA damage and indicates that BS-DRL1 is required to minimize the progressive accumulation of oxidative DNA lesions in the brain. This is particularly important for neurons as the brain is thought to metabolize one fifth of consumed oxygen, and accordingly, ROS is considered a major source of DNA damage in the brain^26^. In fact, it is reported that ROS alone can generate more than 100 different highly mutagenic oxidative base modifications, and increased levels of oxidative DNA damage have been identified in many neurological diseases^25^. A better understanding of this type of genome instability will provide a foundation for studying neuron-specific DDR more generally, which has implications in aging and in a number of neurological diseases caused by mutations in the genes involved in DDR.

## Methods

### Cell culture and primary neuron culture

HEK293 cells (Cat. # CRL-3216) were maintained in high glucose DMEM media (Hyclone) supplemented with 10% heat-inactivated FBS (Biological Industries) and 1% streptomycin/penicillin (Thermo Fisher Scientific) at 37 °C and 5% CO_2_ atmosphere. Primary cortical neurons were cultured as previously described^11^. Briefly, cortical primary neurons from E16 ICR mice were plated on poly-L-lysine-coated 24-well plate (for staining), 6-well plate (for comet assay), 6-cm or 10-cm plates (for biochemistry) in plating media (Neurobasal, supplemented with 10% heat-inactivated FBS, 5 mM Glutamax and 1% penicillin/streptomycin) for 3 h, neurons were maintained in regular media (Neurobasal, 1×B27, supplemented with 5 mM Glutamax and 1% penicillin/streptomycin) at 37 °C and 5% CO_2_ atmosphere. Neurons were infected at DIV (days in vitro) 5 and harvested at DIV9.

### Plasmid and lentivirus generation

The shRNA sequences targeting mouse *BS-DRL1* were cloned into pLKO puromycin or FUGW-H1-GFP-neomycin^40^. Mouse Hmgb1 gene was amplified from primary neurons and cloned into FhsynPW to construct Flag-Hmgb1 overexpression vector. Sequences were provided in Supplementary Table S1 and all the plasmids were verified by Sanger sequencing. To generate lentivirus, expression vectors (pLKO.1 puromycin, FUGW-H1-GFP-neomycin and FhsynPW) and packaging vectors (psPAX2 and pMD2.G) were co-transfected into HEK293T cells at the ratio of 3:2:1. Medium containing lentivirus was collected 48 h later and ultracentrifuged at 20,000 × *g* for 2 h at 4 °C, the pellets were then resuspended in HBSS and stored at −80 °C until use.

### Subcellular fractionation and qPCR

Subcellular fractionation was performed as previously described^41^. Briefly, DIV9 neurons were treated with vehicle or etoposide for 1 h before harvesting. Cytoplasm, nuclear soluble and chromatin were isolated and RNA was extracted with Trizol and reverse transcribed followed by qPCR with Actin, Malat1, and *BS-DRL1* primers. Primers sequences were provided in Supplementary Table S1.

### Comet assay

Alkaline comet assay was performed with Comet assay kit (Trevigen, 4250-050-K) following the manufacturer’s instructions. Briefly, infected primary cortical neurons at DIV9 were treated with vehicle or etoposide for 1 h. Cells at 1 × 10^5^/ml were combined with molten LMAgarose (at 37 °C) at a ratio of 1:10 (v/v) and pipetted onto CometSlide™. After solidifying for 10 min at 4 °C, the slides were immersed in the lysis solution for 1 h at 4 °C and then in freshly prepared alkaline unwinding solution for 1 h at 4 °C in the dark. Slides were then subjected to electrophoresis (21 V for 30 min), washed with ddH_2_O twice for 5 min each, immersed in 75% ethanol for an additional 5 min, stained with SYBR Gold (Thermo Fisher Scientific) for 30 min after drying. Images were taken with fluorescence microscope (Leica) and analyzed with CaspLab software.

### NHEJ reporter assay

The NHEJ reporter assay was performed according to protocols previously reported with minor modification^42^. Briefly, the NHEJ reporter constructs containing pPGK-GFP were digested with HindIII restriction enzymes (Thermo Scientific, #FD0504) and purified using TaKaRa MiniBEST DNA Fragment Purification kit (#9761). Then the linearized construct (1.3 μg) and the transfection control plasmids pPGK-mCherry (0.3 μg) were co-transfected into each well of cultured neurons in 12-well plates with Lipofactamine 2000 (3.2ul). Three days after transfection, cells were prepared and analyzed on FACSVerse™ Flow Cytometer (BD Biosciences) with final data analyzed by the FlowJo software. (Supplementary Fig. 12) The repair efficiency was represented as GFP+/mCherry+.

### Primary neuron staining

Infected primary neurons cultured on glass coverslips were fixed in 4% PFA for 10 min at room temperature and blocked in blocking media (5% normal goat serum, 0.2% Triton X-100 in PBS) for 1 h at room temperature. Cells were incubated with primary and secondary antibodies diluted in antibody dilution media (1% normal goat serum, 0.1% Triton X-100 in PBS) overnight at 4 °C and 1 h at room temperature, respectively, followed by washing four times with PBS. Coverslips were mounted onto glass slides with ProLong™ Gold Antifade Mountant medium containing DAPI (Thermo fisher scientific). Images were taken with Leica SP8 confocal microscope. Image J was used to measure γH2AX signal.

### Histology and Immunofluorescence

*BS-DRL1* knockout or control mice were treated with or without 4Gy gamma-irradiation in the Gammacell 40 Exactor (Nordion, Canada) and recovered for 3 h. Anesthetized mice were perfused transcardially with ice-cold PBS, and 4% PFA, after which brains were incubated in 4% PFA for 24 h (4 °C) and then in 30% sucrose for 48 h (4 °C). Next, serial coronal sections of brains (30 mm thickness) were prepared using a cryostat (Leica). For immunohistochemistry, free-floating sections of the slice were immersed in blocking media (5% normal goat serum, 0.5% Triton X-100 in PBS) for 2 h at room temperature, primary and secondary antibodies diluted in antibody dilution media (2% normal goat serum) overnight at 4 °C and 1 h at room temperature, respectively, followed by washing four times with PBS. Slice were mounted onto glass slides with ProLong™ Gold Antifade Mountant medium containing DAPI (Thermo fisher scientific). Images were taken with Leica SP8 confocal microscope. Image J was used for image analysis.

### TUNEL staining

TdT-UTP nick end labeling (TUNEL) assays were performed with the one step TUNEL kit (Beyotime, C1089) according to the manufacturer’s instructions. Primary neurons were infected at DIV5 and performed TUNEL staining at DIV9.

### ROS detection

Intracellular ROS levels were determined by measuring the oxidative conversion of cell permeable DCFH-DA to fluorescent dichlorofluorescein (DCF) under fluorescence microscope. The assays were performed with the Reactive Oxygen Species Assay Kit (Beyotime, S0033) according to the manufacturer’s instructions.*BS-DRL1* KO or WT primary neurons were cultured for 9 days for ROS detection.

### Western blotting

Primary neurons were harvested and lysed in RIPA buffer with sonication followed by centrifuging for 10 min at 20,000 × g at 4 °C and the supernatant was collected for SDS-PAGE. Membrane was blocked with 5% (m/v) nonfat milk for 1 h at room temperature followed by immunoblotting with indicated primary and secondary antibodies overnight at 4 °C and 1 h at room temperature, respectively. Antibodies are listed in Supplementary Table S2.

### RNA immunoprecipitation (RIP)

For native RIP, DIV9 neurons were lysed in RIPA buffer (50 mM Tris pH 7.5, 150 mM NaCl, 1% NP-40, 0.1% SDS, and 0.5% sodium deoxycholate supplemented with 1× Protease Inhibitor cocktail (Roche) and 100 U of RNA^OUT^ (Thermo fisher scientific) with sonication. 5% of clarified lysates were saved as input and the rest was incubated with antibody-coupled Dynabeads protein G beads at 4 °C for 3 h with rotating. Beads were washed three times with RIPA. RNA was extracted with Trizol (Thermo fisher scientific) following the manufacturer’s instructions. Input and immunoprecipitated RNA was reverse transcribed and the *BS-DRL1* was measured with qPCR. Primer sequences are listed in Supplementary Table S1.

For etoposide treated RIP, neurons were infected with scramble shRNA or *BS-DRL1* shRNA at DIV5 and treated with etoposide for 1 h before subjecting to RIP at DIV9.

### Chromatin binding assay

Chromatin was isolated as previously described^41^. Briefly, neurons were infected with shRNAs at DIV5 and treated with vehicle or etoposide for 1 h at DIV9 before harvesting. 5% neurons were used as input. Input neurons and isolated chromatin were lysed in RIPA buffer with sonication followed by BCA assay and Western blotting. Antibodies are listed in Supplementary Table S2.

### Generation and genotyping of BS-DRL1 knockout mice

All animal experiments were conducted according to protocols approved by the Institutional Animal Care and Use Committee (IACUC) from Interdisciplinary Research Center on Biology and Chemistry (IRCBC), Shanghai Institute of Organic Chemistry, Chinese Academy of Sciences. *BS-DRL1*^−/−^ mice were generated by CRISPR/Cas9 on a C57BL/6N background by Beijing Biocytogen Co., Ltd., Beijing, China. Briefly, in vitro transcribed Cas9 mRNA and two small gRNA were coinjected into mouse zygotes. The sgRNA targeting sequences were 5′-CTG ACC TCT CGG TTT CCT AC-3′ and 5′-TCT GCC ACA GCG ACA CGT CG-3′. The genotyping was determined using PCR with primers provided in Supplementary Table S1.

### RNA isolation and qPCR

Total RNA was extracted with TRIzol (Thermo fisher scientific) following the manufacturer’s instructions and reverse transcribed with Hifair® II 1st Strand cDNA Synthesis SuperMix for qPCR (gDNA digester plus) (Yeason, Shanghai, China). cDNA was quantified using TB Green® Premix Ex Taq™ (Tli RNase H Plus) (Takara) with an Applied Biosystems™ QuantStudio™ 6 Flex Real-Time PCR System (Thermo fisher scientific) following the manufacturer’s instructions.

### Chromatin isolation by RNA purification (ChIRP)

38 20-mer anti-sense DNA probes with 3’BiotinTEG were designed at https://www.biosearchtech.com/support/tools/design-software/chirp-probe-designer and synthesized by GENEWIZ, Inc. (Suzhou, China). Chromatin isolation by RNA purification (ChIRP) was performed as previously described^43^. Briefly, 6 × 10^7^ DIV9 primary neurons were treated with etoposide to induce DNA damage, and then were crosslinked with 1% glutaraldehyde for 10 min at room temperature and lysed in 1 ml lysis buffer (50 mM Tris-HCl, pH 7.0, 10 mM EDTA, 1% SDS, protease inhibitor, PMSF and RNA^OUT^) followed by sonication for 20 min, 2 ml hybridization buffer (50 mM Tris-HCl, pH 7.0, 750 mM NaCl, 1% SDS, 1 mM EDTA, 15% formamide, protease inhibitor, PMSF and RNA^OUT^) was added to the supernatant after centrifugation. Probes were added to the final concentration 50 nM, shaking overnight at 37°C for hybridization, 100 μl washed Dynabeads™ MyOne™ Streptavidin C1 (Thermo fisher scientific) beads were added to each tube for additional 1 h with shaking at 37 °C. Then beads were subjected to five washes with wash buffer (2× SSC, 0.5% SDS, protease inhibitor, PMSF and RNA^OUT^). RNA was extracted from beads and input with Trizol followed by reverse transcription and qPCR. Proteins were eluted twice with elution buffer (2% trifluoroacetic acid (TFA) and 50% acetonitrile) at 50 °C with shaking and subjected to MS analysis.

### 3′RACE

3′RACE was performed with SMARTer® RACE 5′/3′ Kit (Takara) following the manufacturer’s instructions. In brief, total RNA extracted from primary neurons with TRIzol (Thermo fisher scientific) was used for first-strand cDNA synthesis, then touchdown PCR was used to amplify different transcripts. PCR products were gel extracted and cloned into linearized pRACE vector, sequences were determined with Sanger sequencing.

### RNAscope

We designed a 20 ZZ probe targeting 2599-3591 of NR_040311.1. The probe cross detects transcript variants 1747,3404,13049 and 3456.

12-month-old WT B6N/C57 mice were treated with 12 Gy X-ray or not and the *BS-DRL1* FISH was performed with RNAscope® Multiplex Fluorescent Reagent Kit v2 following the manufacturer’s instructions.

*BS-DRL1* in situ hybridization with immunofluorescence (IF) was also performed following the manufacturer’s instructions of RNAscope® Multiplex Fluorescent v2 Assay combined with IF and we modified the RNAscope protocol to enable IF of γH2AX, NeuN, calbindin, and PV with Alexa Fluor® secondary antibody. Specifically, prior to the RNAscope assay, bake the slides at 60 °C for 2 h in a dry oven. We then pre-treated slides according to RNAscope® protocol. We performed single probe FISH to detect *BS-DRL1* according to the manufacturer’s instructions followed by IF. The primary antibodies were diluted in a lower dilution factor(0.5 times compared to the directly IF) and incubate 12 h or overnight at 4 °C. The secondary antibody was incubated for 2 h at RT. Finally, mount the slides as protocol. The images were taken by Olympus VS200.

### Behavior test

#### Open field test

Before starting the test, mice were placed in the testing room to acclimate for 30 min. Individual mouse was then placed in the open-field box (40 cm × 40 cm) and allowed it to explore for 30 min under dim lighting. The paths of mice were recorded with a video camera and analyzed using Etho Vision XT (Noldus). The box was divided into a central field (24 cm × 24 cm) and the periphery.

#### Grip strength

The grip strength of mice was measured using a grip strength meter (Bioseb, USA). Mice were held by the base of the tail close to the wire mesh pull-bar and allow them to reach and grab the bar with their forelimbs or all four limbs. Mice were then pulled away from the bar until their grip was released. Repeat twice and the highest force was recorded of three times. Three trials were performed for each mouse.

#### Pole test

Mice were placed head-up on the upper end of a vertical and rough-surfaced pole (Diameter: 1 cm; length: 50 cm). The time to orient downward completely and total time to descend were recorded. The maximum time allowed was 120 s. After a trail of training, mice receive four test trials.

#### Rotarod test

Motor coordination and balance were evaluated using the accelerating rotarod test with a 3 cm diameter rod. Mice were first trained 3 days (3 trials per day). Mice were placed on the rod and allowed them to walk on the rod rotating at a constant speed (5 rpm/min) for 2 min at first two trials and accelerating speed (accelerate from 5 to 40 rpm/min in 90 s and then hold at constant speed) for 2 min at third trial. On day 4, each mouse was tested at accelerating speed (accelerate from 5 to 40 rpm/min in 90 s and then hold at constant speed) for 5 min on three independent trials. The latency of the mice to fall off the rod was recorded and data from there trials were averaged.

#### Gait analysis

Quantitative gait analysis of locomotion was performed using a GaitLab apparatus for rodents (ViewPoint, France), based on the CatWalk method^44^. The system consists of a transparent 125 cm long runway illuminated by a fluorescent tube. When an object (i.e., rat paws) touches the surface, the contact area lights up, detected by a video camera positioned underneath that monitoring the steps of the animal across the runway. To record baseline scores, animals walk across the runway until they voluntarily performed the exercise (3–4 correct crossings), and the most regular crossings were selected for footprint analysis by dedicated Viewpoint software.

### Golgi staining

Golgi staining was performed with FD Rapid GolgiStain^TM^ Kit (PK401A) according to the manufacturer’s instructions. 12 m WT and *BS-DRL1* KO mice were Anesthetized and quickly decapitate the head. Immerse the freshly harvested brains into solution A and B mixture prepared 24 h prior to use. Two weeks later, transfer brains into solution C, and store at room temperature in the dark for 72 h. Freeze tissue and cut 100 μm sections. Stain sections and mount with Neutral Balsam Mounting Medium. The images were taken by Olympus VS200 and performed Z min projection. ImageJ was used for sholl analysis.

### CHIP and qPCR

The primary cortex neurons were infected with *BS-DRL1* shRNA or Scr-shRNA virus at DIV3 for 48 h, and lentivirus expressing mCherry-I-PpoI was transducted into the neurons that *BS-DRL1* were KD or not for 48 h. Cells were treated with ETO for 1 h and crosslinked by adding formaldehyde (1% final concentration) followed by quenching with glycine (Sigma) at 0.125 M final concentration, then harvested. ChIP-Grade Protein G Magnetic beads (Thermo Fisher Scientific, 10004D) were subjected to antibodies indicated (γH2AX,HMGB1 and p-DNA-PKcs), next day, cell pellets were resuspended in cell lysis buffer (10 mM pH 8.0 Tris-Cl, 10 mM NaCl, 0.5% NP-40, 1× proteinase inhibitor cocktail) and incubate on ice for 20 min, discard the supernatant and resuspend pellet with Nuclear Lysis buffer, then the cell lysate were sonicated. In all, 10% input was aliquoted separately. The DNA-protein immunocomplexes were pull down by beads-antibody complex and subjected to serial washes buffers of RIPA-150 (50 mM pH 8.0 Tris-HCl, 0.15 M NaCl, 1 mM EDTA, pH 8, 0.1% SDS, 1% Triton X-100, 0.1% sodium deoxycholate), RIPA-500 (50 mM Tris-HCl, pH 8, 0.5 M NaCl, 1 mM EDTA, pH 8, 0.1% SDS, 1% Triton X-100, 0.1% sodium deoxycholate), LiCl (50 mM Tris-HCl, pH 8, 1 mM EDTA, pH 8, 1% Nonidet P-40, 0.7% sodium deoxycholate, 0.25 M LiCl_2_), TE buffer (10 mM Tris-HCl, pH 8, 1 mM EDTA, pH 8). Then the crosslinked DNA was eluted with Elution buffer (1% SDS + 100 mM NaHCO3) at 65°C. Subsequently, the samples including input were incubated with RNase A and proteinase K overnight, the DNA was eluated with QIAquick PCR Purification Kit (Qiagen) as per the manufacturer’s instructions.

Occupancy of the DNA-bound proteins was measured by subjecting eluted DNA to qPCR using an Applied Biosystems™ QuantStudio™ 6 Flex Real-Time PCR System (Thermo fisher scientific) with primers for chrom5 I-PpoI site^45^. Primers are listed in Supplementary Table S1. The percentage of input was calculated by dividing the amount of DNA obtained from the IP of the given factor by the total amount of DNA (input) and normalized for background signal (non-specific IgG control). Each experiment was carried out in triplicate and the results were analyzed using the ΔΔCt method.

### WGS

Genomic DNA was extracted from WT or *BS-DRL1* knockout mice with AllPrep DNA/RNA Mini Kit (Qiagen, cat#80204) following the manufacturer’s instructions. DNA concentrations were measured with the NanoDrop 2000 (Thermo Fisher Scientific), and sheared with Covaris S220 Sonicator (Covaris) to about 350 bp. Fragmented DNA was purified using Sample Purification Beads (Illumina). Libraries were prepared with the TruSeq Nano DNA Sample Prep Kits (Illumina) according to the manufacturer’s instructions. Concentration and size distribution of the enriched DNA libraries was analyzed with the Qubit 2.0 fluorometer dsDNA HS Assay (Thermo Fisher Scientific) and Agilent BioAnalyzer 2100 (Agilent), respectively. Paired-end sequencing is performed in Illumina HiSeq 10 with 2×150 paired-end in WuXi NextCODE at Shanghai, China. Read sequences were mapped by Burrows-Wheeler Aligner (BWA)^46^ to the mouse reference genome (mm10). Single Nucleotide Variants (SNV) and InDel were analyzed with Sentieon^47^. Copy number variation and structural variation were determined with CNVkit^48^ and Manta^49^.

The off-target screen was performed with rules of ±500 bp from the site of PAM within the predicted off-target genes from website of http://crispor.tefor.net/.

### Statistical analysis

The statistical analysis was performed using GraphPad Prism 6 software. Two-tailed Student’s *t* tests was used with 95% confidence intervals. All results were expressed as mean ± SD or mean ± SEM with *P* < 0.05 indicating significance.

### Reporting summary

Further information on research design is available in the Nature Research Reporting Summary linked to this article.

## Supplementary information

Supplementary Information

Reporting Summary

## Data Availability

The experiment data that support the findings of this study are available from the corresponding author upon reasonable request. The GenBank accession numbers of new transcripts of BS-DRL1 are MW969901, MW969902, MW969903, MW969904, MW969905. The analysis of active histone marks on the BS-DRL1 gene locus (Supplementary Fig. 1f) was perform with public dataset (accession code: GSE29184). Source data are provided with this paper.

## Source data

Source Data
