# Supplementary material for: Long noncoding RNA BS-DRL1 modulates the DNA damage response and genome stability by interacting with HMGB1 in neurons
Source: Nat Commun. 2021 Jul 1;12:4075. doi: 10.1038/s41467-021-24236-z (PMC8249382; doi:10.1038/s41467-021-24236-z)
Supplement: Supplementary file 1 — Supplementary Information [file 41467_2021_24236_MOESM1_ESM.pdf]

Supplemental information

Long noncoding RNA BS-DRL1 modulates the DNA damage response and genome stability by interacting with HMGB1 in neurons

Min-Min Lou, Xiao-Qiang Tang, Guang-Ming Wang, Jia He, Fang Luo, Mingfeng Guan, Fei Wang, Huan Zou, Junying Wang, Qun Zhang, Ming-jian Xu, Qi-Li Shi, Li-Bing Shen, Guo-Ming Ma, Yi Wu, Yao-Yang Zhang, Ai-bin Liang, Ting-Hua Wang, Liu-Lin Xiong, Jian Wang, Jun Xu, Wen-Yuan Wang

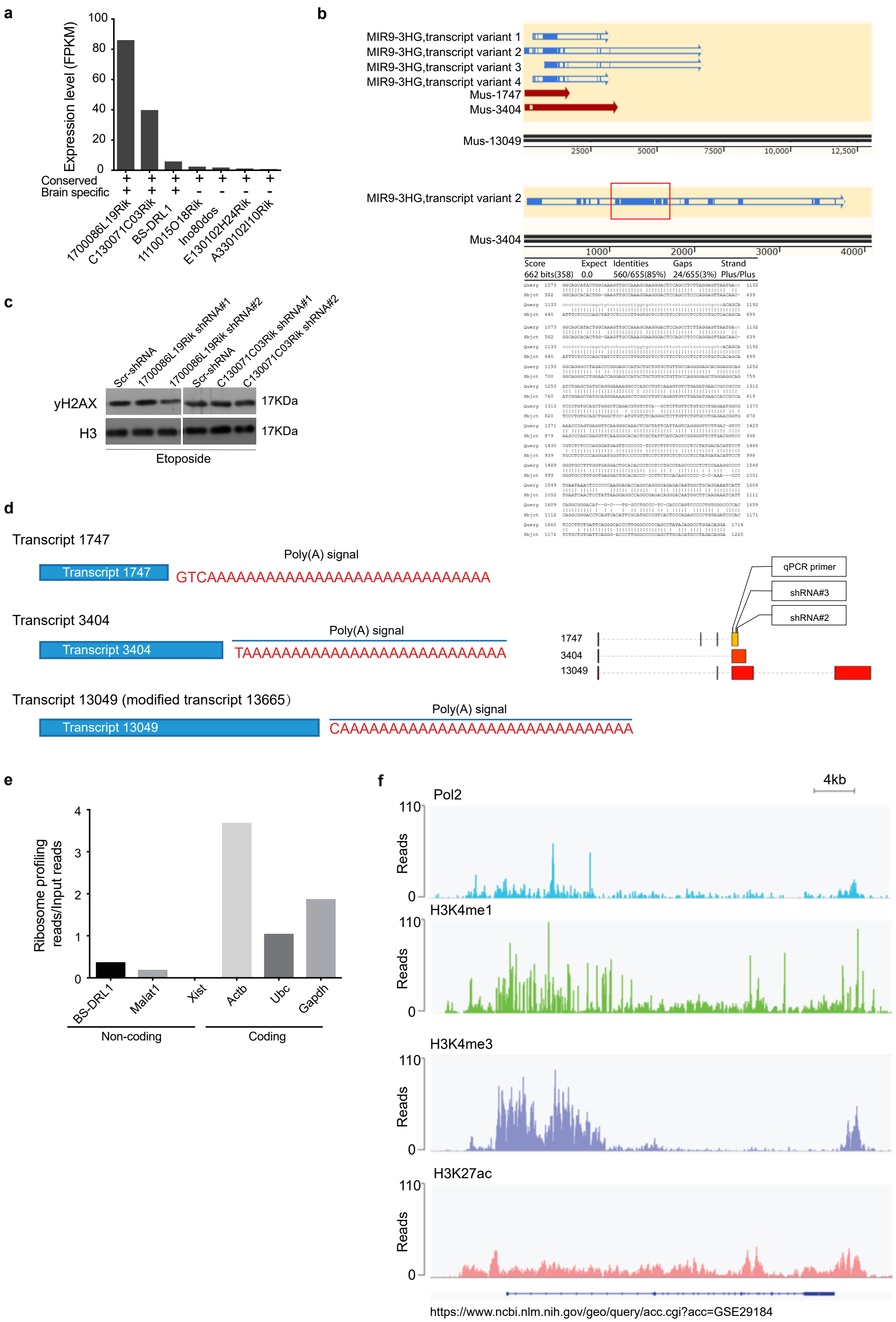
a
Expression level (FPKM)
100
80
60
40
20
0
Conserved + + + + + + +
Brain specific + + + - - - -
1700086L19Rik
C130071C03Rik
BS-DRL1
1110015O18Rik
Ino80dos
E130102H24Rik
A330102I10Rik
b
MIR9-3HG,transcript variant 1
MIR9-3HG,transcript variant 2
MIR9-3HG,transcript variant 3
MIR9-3HG,transcript variant 4
Mus-1747
Mus-3404
Mus-13049
2500
5000
7500
10,000
12,500
MIR9-3HG,transcript variant 2
Mus-3404
1000
2000
3000
4000
Score Expect Identities Gaps Strand
662 bits(358) 0.0 560/655(85%) 24/655(3%) Plus/Plus
c
Scr-shRNA
1700086L19Rik shRNA#1
1700086L19Rik shRNA#2
Scr-shRNA
C130071C03Rik shRNA#1
C130071C03Rik shRNA#2
yH2AX
H3
17KDa
17KDa
Etoposide
d
Transcript 1747
Poly(A) signal
Transcript 1747
GTCAAAAAAAAAAAAAAAAAAAAAAAAAAAAAAA
Transcript 3404
Poly(A) signal
Transcript 3404
TAAAAAAAAAAAAAAAAAAAAAAAAAAAAA
Transcript 13049 (modified transcript 13665)
Poly(A) signal
Transcript 13049
CAAAAAAAAAAAAAAAAAAAAAAAAAAAAAAAA
qPCR primer
shRNA#3
shRNA#2
1747
3404
13049
e
Ribosome profiling reads/Input reads
4
3
2
1
0
BS-DRL1
Malat1
Xist
Actb
Ubc
Gapdh
Non-coding
Coding
f
4kb
Pol2
110
Reads
0
H3K4me1
110
Reads
0
H3K4me3
110
Reads
0
H3K27ac
110
Reads
0
https://www.ncbi.nlm.nih.gov/geo/query/acc.cgi?acc=GSE29184

Supplementary Figure1. Characterization of BS-DRL1.

a. Conservation and brain specificity of uncharacterized lncRNAs that are expressed in primary neurons (FPKM≥1).

b. Homologous sequence alignment of BS-DRL1 between mouse and human. Four transcript variants of human MIR9-3HG were aligned to three highly expressed mouse ones with Snapgene software (up). Human transcript variant 2 was aligned to mouse 3404 transcripts with Snapgene (median) and the detailed aligned sequences were displayed (down).

c. Western blot analysis of γH2AX level in primary neurons infected with lentivirus expressing indicated shRNAs virus. Neurons were treated with vehicle or ETO for 1 hour, lysed immediately and then for western blot analysis. n = 3 biologically independent samples.

d. Verification of three newly annotated transcripts with RACE (Rapid amplification of cDNA ends). See “data availability” for the detailed sequencing information (left) and schematic illustration of BS-DRL1 shRNA and qPCR primer site on the 1747, 3404 and 13049 transcripts (right).

e. Coding potential assessed with ribosome profiling. Malat1 and Xist were used as noncoding RNA controls whereas Actb, Ubc and Gapdh were used as coding RNA controls. This data was generated by analyzing the data from Gonzalez, Christian, et al. "Ribosome Profiling Reveals a Cell-Type-Specific Translational Landscape in Brain Tumors", The Journal of Neuroscience 34.33(2014):10924-10936.

f. Schematic illustration of ChIP profiling of Pol II and active histone marks on the BS-DRL1 gene locus (from ENCODE).
(https://www.ncbi.nlm.nih.gov/geo/query/acc.cgi?acc=GSE29184).

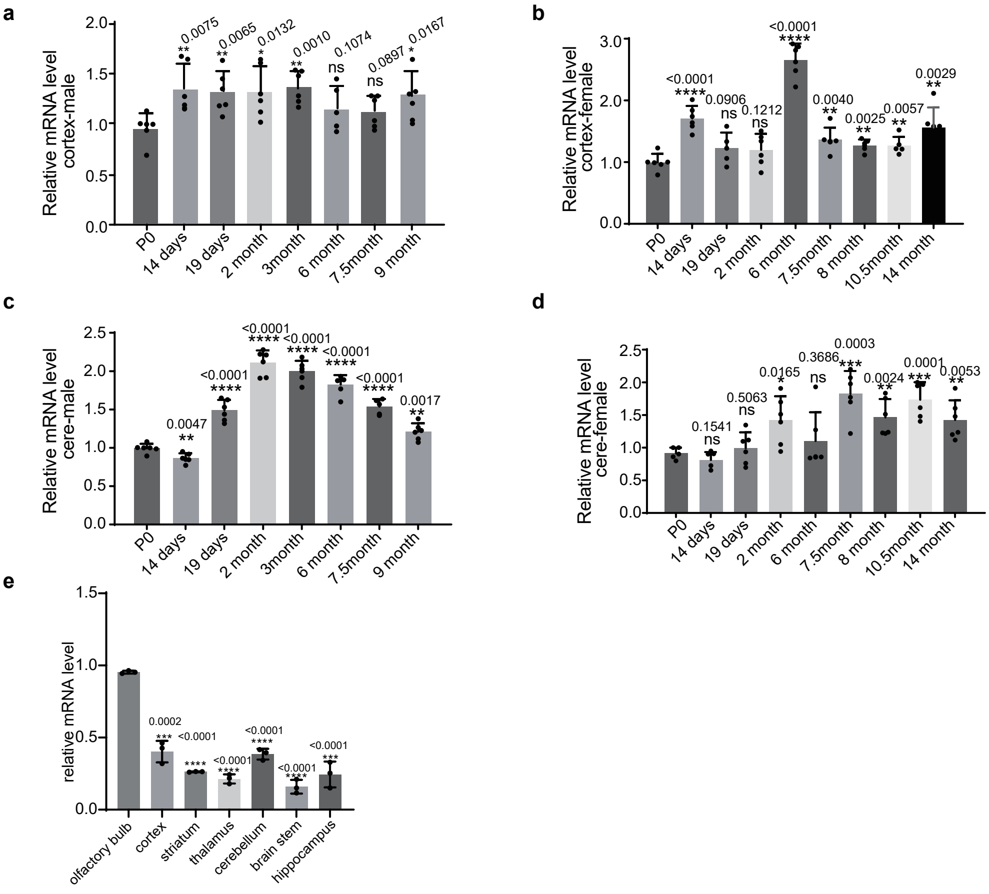

Supplementary Figure2. BS-DRL1 expression pattern in mouse brain.

a-d. Expression level of BS-DRL1 at different age of C57BL/6N mice. n=3, all data were compared to P0. Data are presented as mean ± SD. *p<0.05, **p<0.01, ***p<0.001, ****p<0.0001.

e. Expression level of BS-DRL1 in different brain sub-regions. Data are presented as mean ± SD. n=3. *p<0.05, **p<0.01, ****p<0.0001.

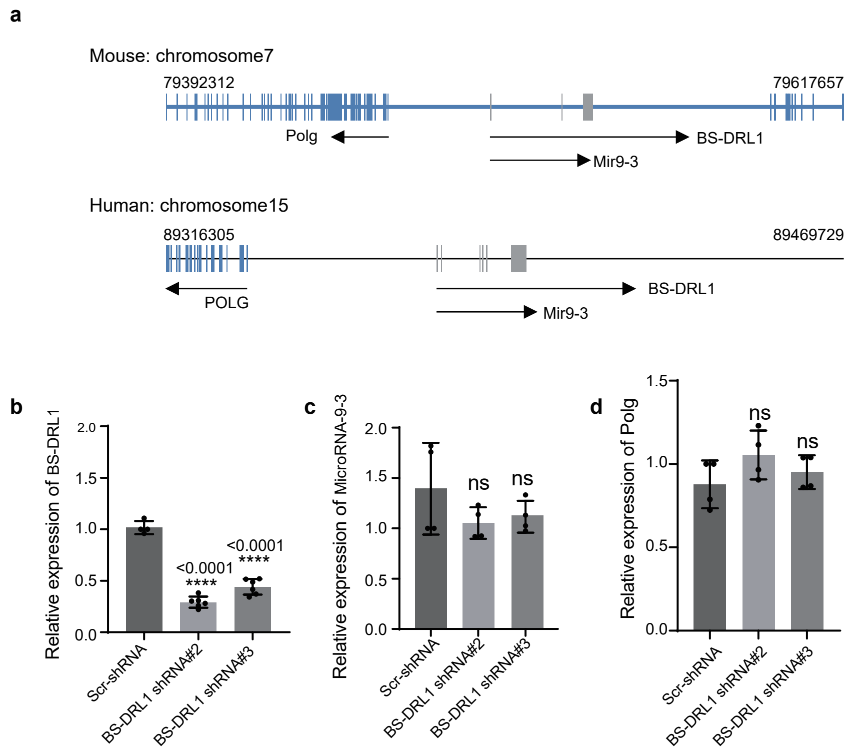

Supplementary Figure3. BS-DRL1 doesn't affect Polg or miR-9-3 expression.
a. Location of BS-DRL1 and its neighboring genes in mouse and human genome. miR-9-3 is encoded by intron 1 of BS-DRL1, Polg is located upstream of BS-DRL1.
b. Verification of the shRNAs used in this study. Different shRNAs targeting BS-DRL1 virus were introduced into neurons for 96 hours and RNA was extracted for qPCR analysis. Data are presented as mean ± SD. ****p<0.0001. n =4-6 biologically independent samples.
c, d. The expression level of Polg and miR-9-3 in BS-DRL1 depleted neurons. Primary neurons were infected with lentivirus expressing BS-DRL1 shRNAs, and miR-9-3 (c) and Polg (d) were quantitated by RT-qPCR 4 days later. Data are presented as mean ± SEM. Two-tailed Student's t tests. ns: not significant. n =4 biologically independent samples.

a

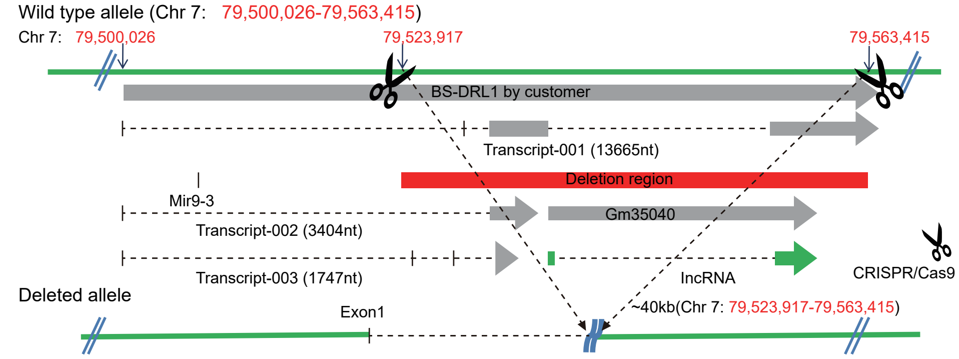

b

	Seq	NO. of predicted off-target	Off-target find in WGS
sgRNA2	CTGACCTCTCGGTTTCCTAC	107	0
sgRNA8	TCTGCCACAGCGACACGTCG	29	0

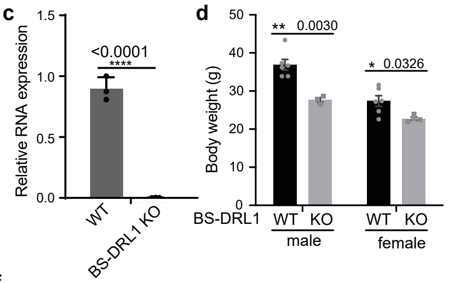

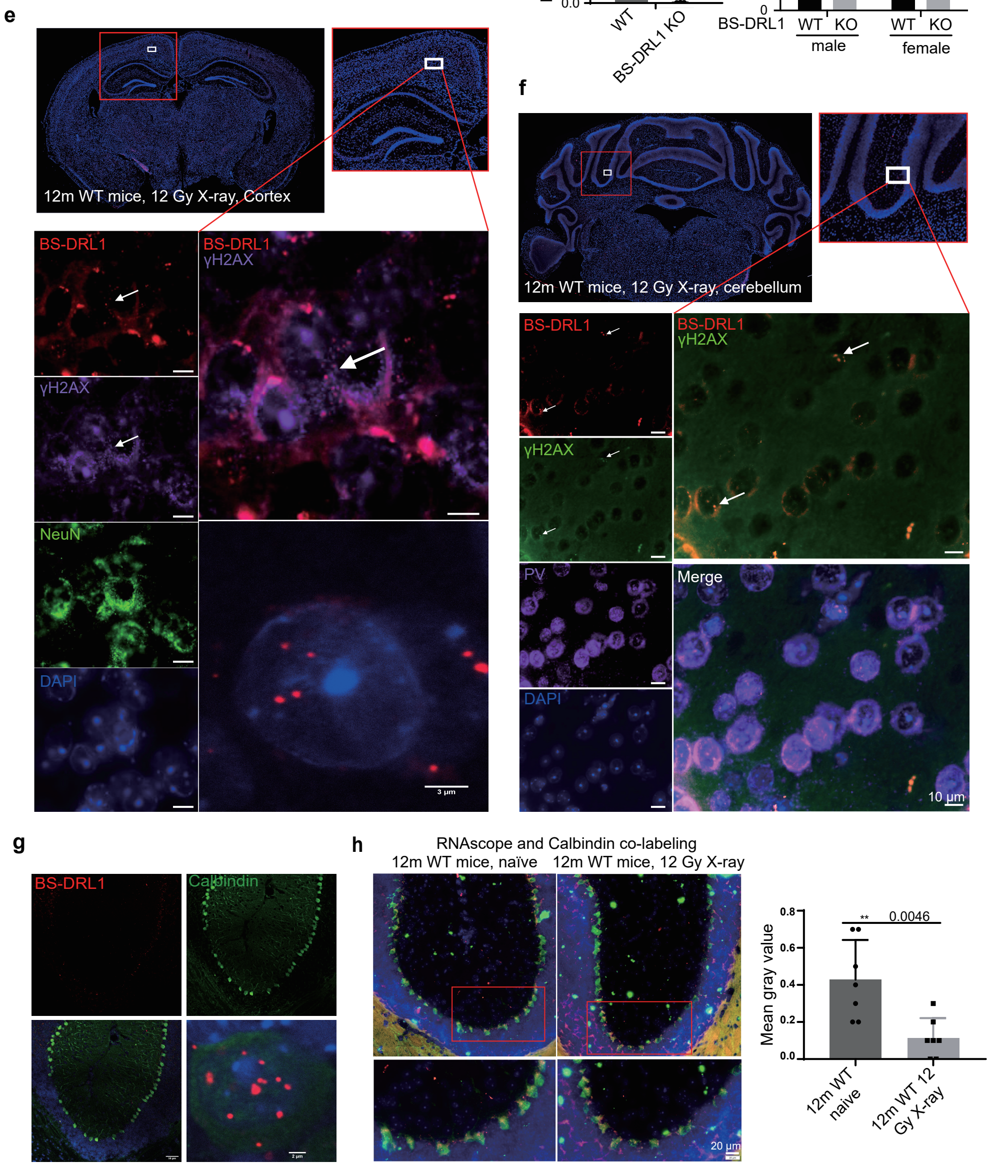

i

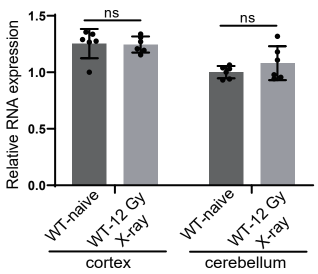

Supplementary Figure4. Generation of BS-DRL1 KO mice and BS-DRL1 in situ.

a. Strategy for generating BS-DRL1 knockout mice. Based on our RNA-Seq and RACE data, genomic DNA in Chr 7: 79,523,917- 79,563,415 was deleted.

b. Off target analysis of sgRNAs used for generating BS-DRL1 knockout mice by crispr-cas9, and ± 500bp from PAM were screened for off target.

c. Verification of the BS-DRL1 KO mice used in this study by RT-qPCR. Brain tissues of WT and BS-DRL1 knockout mice were collected for qPCR analysis. Data are presented as mean ± SD. n=3.

d. BS-DRL1 KO mice are smaller and weigh less compared to the littermate controls. Data are presented as mean ± SEM. Male, n=6:3; female, n=6:4. *p<0.05, ***p<0.001.

e, f. BS-DRL1 in situ with RNAscope probe (red)and immunofluorescence with NeuN, PV and γH2AX on 12m WT mice treated with 12 Gy X-ray. Cortex (left) and cerebellum (right). n = 3 biologically independent experiments.

g. BS-DRL1 in situ with RNAscope probe (red) and immunofluorescence with calbindin using 12 Gy X-ray treated mice. n = 3 biologically independent experiments.

h. BS-DRL1 in situ with RNAscope probe and immunofluorescence with calbindin on 12m WT mice treated with 12 Gy X-ray or not. n=3 mice and 7 views of the slices, Data are presented as mean ± SD,**p<0.01.

i. mRNA expression level of BS-DRL1 in cortex and cerebellum between 6m WT and WT 12 Gy X-Ray treated mice. Data are presented as mean ± SD. n=3. ns: not significant.

.

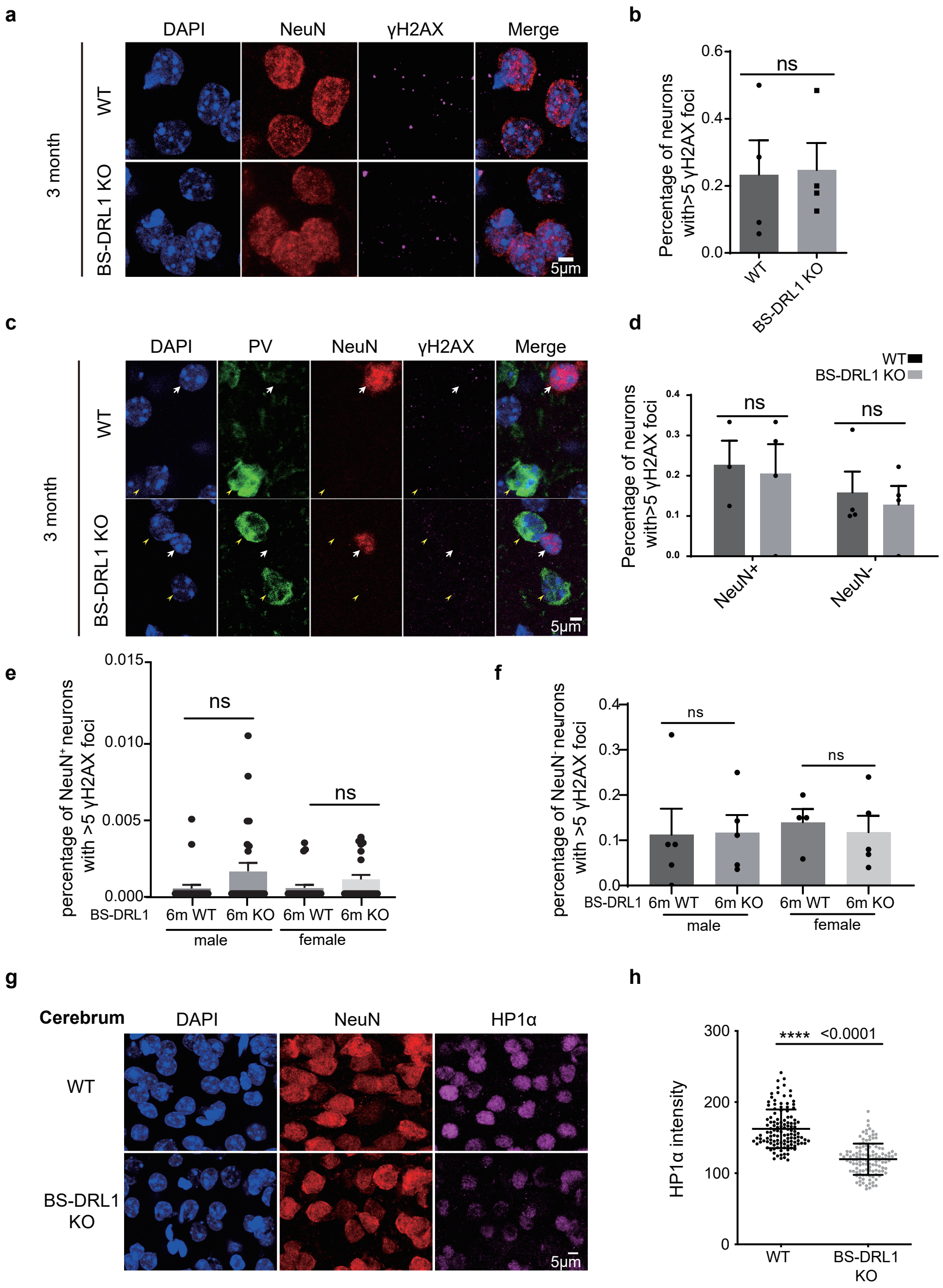

Supplementary Figure5. DNA damage in 3- and 6-month-old mice.

a-d. Representative images and statistical analysis of DNA damage in cerebral cortex and cerebellum sections from 3-month-old naïve mice. Immunofluorescence staining was performed with γH2AX, NeuN and parvalbumin antibodies. White arrows indicate NeuN+ neurons, yellow arrowheads indicate PV neurons. Scale bar: 5 μm. Data are presented as mean ± SD. For each group, more than 100 neurons were counted from 3 mice. ns: not significant.

e, f. Quantification of DNA damage level in NeuN+ neurons of cerebral cortex and NeuN- neurons of cerebellum sections from 6-month-old male and female BS-DRL1 KO mice or littermate controls. Neurons and DNA damage were labeled with NeuN and γH2AX antibodies, respectively. Data are presented as mean ± SD. For each group, more than 100 neurons were counted from 3 mice. ns: not significant.

g, h. Representative images and quantification of HP1α staining in cerebral cortex of gamma-irradiation treated 3-month-old mice. Scale bar: 5 μm. Data are presented as mean ± SD. For each group, more than 200 neurons were counted from 3 mice. ****$p<0.0001$.

a

SNPs/InDels mutations in KO mice

Type of mutation	Count
Total number	12149
dbSNP	1161 (9.56%)
UTR3	277
UTR5	47
upstream	57
downstream	70
upstream, downstream	3
exonic	3039
splicing	7
unknown	34
intergenic	5465
intronic	2776
frameshift insertion	11
frameshift deletion	10
nonframeshift deletion	1
ncRNA_exonic	124
ncRNA_intronic	281

b

WGS

Sample	InDel	SNP	total
WT1	127081	63537	190618
WT2	129836	61592	191428
WT3	129203	64219	193422
KO1	126940	49897	176837
KO2	127511	50020	177531
KO3	133484	51980	185464

c

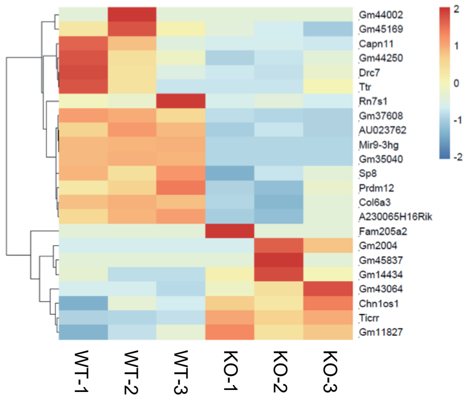

d

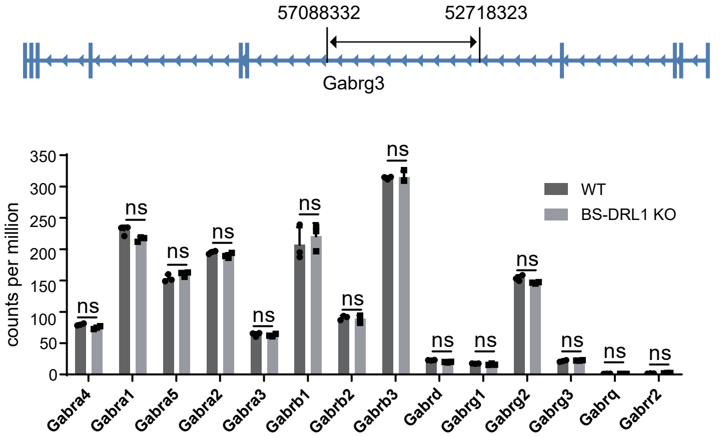

Supplementary Figure6. Summary of WGS and RNA-Seq data of BS-DRL1 KO mice.

a. Summary of mutations and Indels identified in BS-DRL1 KO mice.
b. Detailed WGS information of individual WT and BS-DRL1 KO mice.
c. Heatmap of differentially expressed genes identified by RNA-seq in BS-DRL1 KO mice brain tissues compared with the littermate controls.
d. A DNA fragment in the intron region of Gabrg3 was deleted in BS-DRL1 KO mice, but it does not affect the expression level of Gabrg3 and other Gabr family members (RNA-seq data). Data are presented as mean ± SD, n=3, ns: not significant.

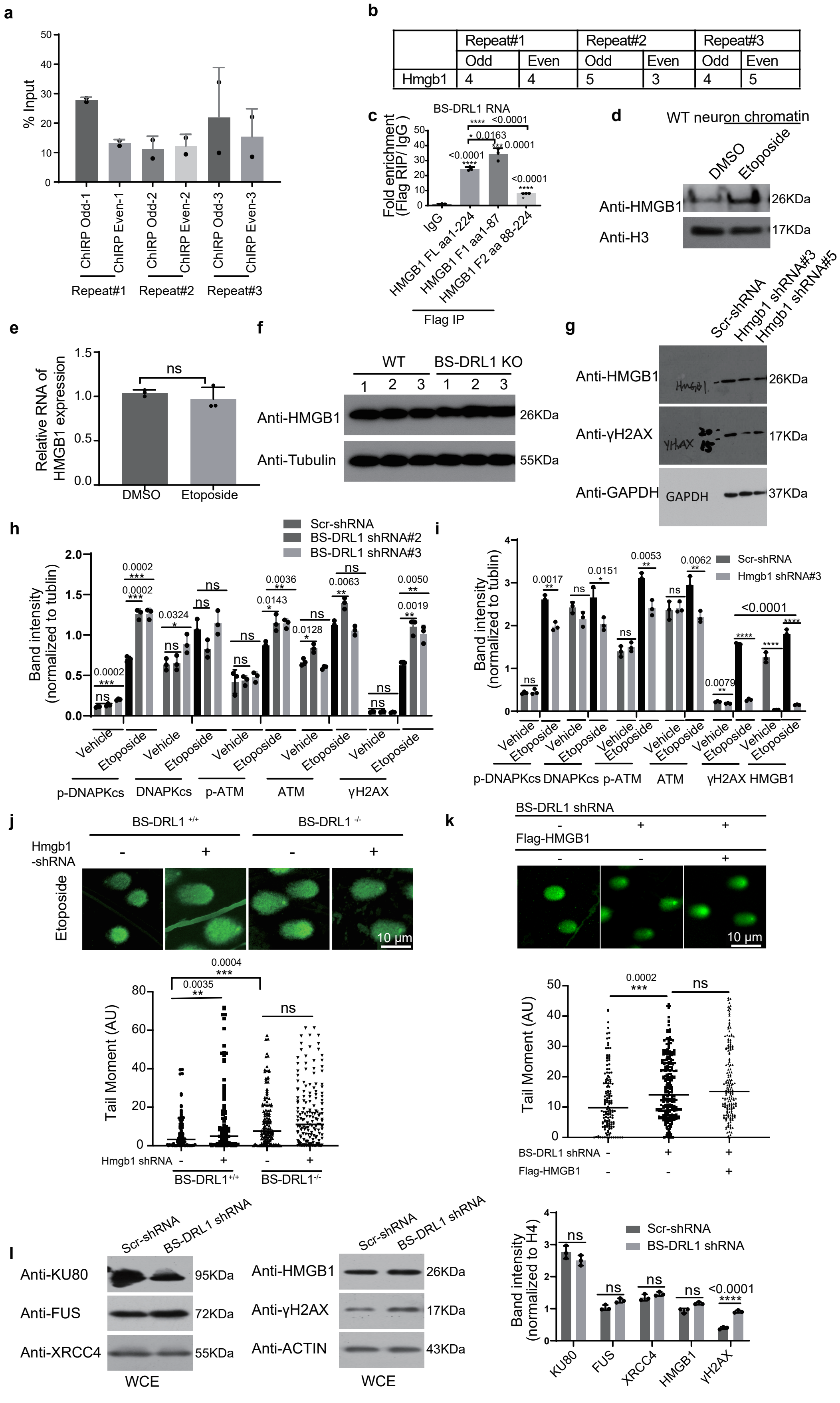
a
% Input
ChIRP Odd-1
ChIRP Even-1
ChIRP Odd-2
ChIRP Even-2
ChIRP Odd-3
ChIRP Even-3
Repeat#1
Repeat#2
Repeat#3
b
Repeat#1 Repeat#2 Repeat#3
Odd Even Odd Even Odd Even
Hmgb1 4 4 5 3 4 5
c
BS-DRL1 RNA
Fold enrichment (Flag RIP/ IgG)
IgG
HMGB1 FL aa1-224
HMGB1 F1 aa1-87
HMGB1 F2 aa 88-224
Flag IP
d
WT neuron chromatin
DMSO
Etoposide
Anti-HMGB1
Anti-H3
26KDa
17KDa
e
Relative RNA of HMGB1 expression
ns
DMSO
Etoposide
f
WT
BS-DRL1 KO
Anti-HMGB1
Anti-Tubulin
26KDa
55KDa
g
Scr-shRNA
Hmgb1 shRNA#3
Hmgb1 shRNA#5
Anti-HMGB1
Anti-γH2AX
Anti-GAPDH
26KDa
17KDa
37KDa
h
Scr-shRNA
BS-DRL1 shRNA#2
BS-DRL1 shRNA#3
Band intensity (normalized to tublin)
Vehicle
Etoposide
p-DNAPKcs
DNAPKcs
p-ATM
ATM
γH2AX
i
Scr-shRNA
Hmgb1 shRNA#3
Band intensity (normalized to tublin)
Vehicle
Etoposide
p-DNAPKcs
DNAPKcs
p-ATM
ATM
γH2AX
HMGB1
j
BS-DRL1 +/+
BS-DRL1 -/-
Hmgb1 -shRNA
Etoposide
10 μm
Tail Moment (AU)
Hmgb1 shRNA
BS-DRL1+/+
BS-DRL1-/-
k
BS-DRL1 shRNA
Flag-HMGB1
10 μm
Tail Moment (AU)
BS-DRL1 shRNA
Flag-HMGB1
l
Scr-shRNA
BS-DRL1 shRNA
Anti-KU80
Anti-FUS
Anti-XRCC4
95KDa
72KDa
55KDa
WCE
Anti-HMGB1
Anti-γH2AX
Anti-ACTIN
26KDa
17KDa
43KDa
WCE
Band intensity (normalized to H4)
KU80
FUS
XRCC4
HMGB1
γH2AX

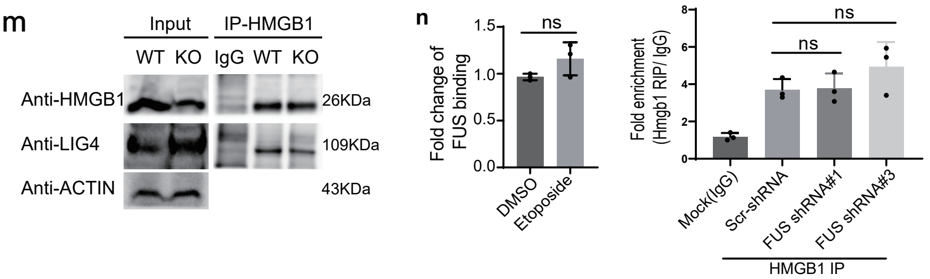

Supplementary Figure7. BS-DRL1 interact with HMGB1 to regulate DDR.

a. Identifying proteins binding with BS-DRL1 by ChIRP, Data are presented as mean ± SEM from 3 replicates.

b. Number of HMGB1 peptides identified in each ChIRP-MS repeat.

c. Functional domains of HMGB1 displayed different affinity with BS-DRL1. Virus expressing Flag-HMGB1 full length (aa1-224), Flag-F1 (aa1-87) and Flag-F2 (aa88-224) were introduced into DIV7 neurons, 48 hours later, cells were treated with ETO for 1 hour before harvested for Flag RIP. Data are presented as mean ± SD. n=3, *p<0.05, ***p<0.001, ****p<0.0001.

d. The assembly of HMGB1 on chromatin was enhanced by ETO treated. DIV9 primary cortical neurons were treated with ETO or vehicle for 1 hour and processed for fractionation and western blot analysis. n =3 biologically independent samples.

e. HMGB1 was not affected by the ETO treatment. DIV9 WT primary neurons were treated with ETO or vehicle for 1 hour and harvested immediately for RNA analysis by RT-qPCR. Data are presented as mean ± SD. ns: not significant. n=3.

f. Protein level of HMGB1 in 9-month-old naïve mice brain was evaluated by western blotting analysis. n =3 biologically independent samples.

g. The original gel image of fig 4e. n =3 biologically independent samples.

h. i. Quantifications of figure 5a (h) and 5e (i). n=3, Data are presented as mean ± SD. ns: not significant, *p<0.05,**p<0.01, ***p<0.001, ****p<0.0001.

j. HMGB1 KD based on the BS-DRL1 knockout did not exacerbate the neuron DNA damage. Primary cortical neurons from WT or BS-DRL1 KO mice were infected with HMGB1-shRNA or Scr-shRNA virus and treated with ETO for 1 hour and harvested for comet assays. Tail moment was analyzed with CaspLab software. Scale bar: 10 µm. Data are presented as mean ± SD. **p<0.01, ***p<0.001, ns: not significant. AU: arbitrary units. n=96 cells.

k. HMGB1 could not rescue the DNA damage caused by BS-DRL1 deficiency. Primary cortical neurons from WT or BS-DRL1 KO mice were infected with flag or flag-HMGB1 virus and treated with ETO for 1 hour followed by comet assays. Tail moment was analyzed with CaspLab software. Scale bar: 10 µm. Data are presented as mean ± SD. ***p<0.001, ns: not significant. AU, arbitrary units. n = 142 cells.

l. Expression levels of HMGB1 and other DNA-damage-related proteins in whole cell extract of BS-DRL1 KD neurons. Primary neurons infected with lentivirus expressing BS-DRL1-shRNA or Scr-shRNA were treated with ETO for 1 hour and harvested for western blotting analysis. Data are presented as mean ± SD. n=3, ***p<0.001, ns: not significant. (right)

m. BS-DRL1 deficiency disturbed the interaction of HMGB1 and LIG4. 12-month-old WT and BS-DRL1 KO mice were treated with 12 Gy X-ray and the brain were lysed for HMGB1 IP. n =3 biologically independent experiments.

n. Fold change of BS-DRL1 binds to FUS validated by HITS-CLIP (left). Primary neurons were treated with ETO or DMSO for 1 hour and processed for HITS-CLIP analysis. And RIP-(RT-qPCR) showed no difference between Scr-shRNA and FUS shRNAs on the interaction of HMGB1 and BS-DRL1 in neurons (right). Primary neurons transfected with indicated virus were treated with etoposide for 1 hour and harvested for HMGB1 RIP. Data are presented as mean ± SD. n = 3. ns: not significant.

.

a

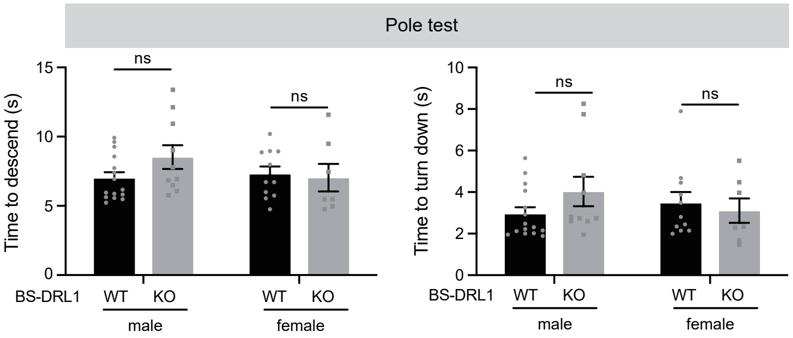

b

Parameters	Unit	male BS-DRL1 KO Vs.WT fl	fr	hl	hr	female BS-DRL1 KO Vs.WT fl	fr	hl	hr
Stride Length	(cm)	ns	ns	ns	ns	ns	ns	ns	ns
Stride Frequency	(steps/s)	ns	ns	ns	ns	↓ p=0.0174	↓ p=0.0226	↓ p=0.0092	ns
Swing time	(s)	ns	ns	ns	ns	ns	↓ p=0.0438	ns	ns
Stance time	(s)	ns	ns	ns	ns	ns	↓ p=0.0452	↓ p=0.0104	ns
area	(cm2)	ns	ns	ns	ns	ns	ns	ns	ns
width	(cm)	ns	ns	ns	ns	ns	ns	ns	ns
length	(cm)	ns	ns	ns	ns	ns	ns	ns	ns
mean intensity sum		ns	ns	ns	ns	↓ p=0.0065	↓ p=0.0494	ns	↓ p=0.0327
fore gap	(cm)	ns	NA	NA	NA	ns	NA	NA	NA
hind gap	(cm)	NA	NA	ns	NA	NA	NA	ns	NA
left pair gap	(cm)	ns	NA	NA	NA	ns	NA	NA	NA
right pair gap	(cm)	NA	ns	NA	NA	NA	ns	NA	NA
fore lag	(cm)	ns	NA	NA	NA	ns	NA	NA	NA
hind lag	(cm)	NA	NA	ns	NA	NA	NA	ns	NA
left pair lag	(cm)	ns	NA	NA	NA	ns	NA	NA	NA
right pair lag	(cm)	NA	ns	NA	NA	NA	ns	NA	NA
Duty Factor	(real#)	↓ p=0.0259				↓ p=0.0452			
speed	(cm/s)	ns				ns			

Supplementary Figure8. Pole tests and gait analysis of BS-DRL1 KO mice.

a. No difference was observed in pole test of 6-month-old BS-DRL1 KO mice compared to the littermate controls. Data are presented as mean ± SD. Male, n=15:10; female, n=11:7.

b. The detailed information of gait analysis of 6-month-old BS-DRL1 KO mice compared to the littermate controls. Male, n=15:10; female, n=11:7. ns: not significant, NA, not available.

.

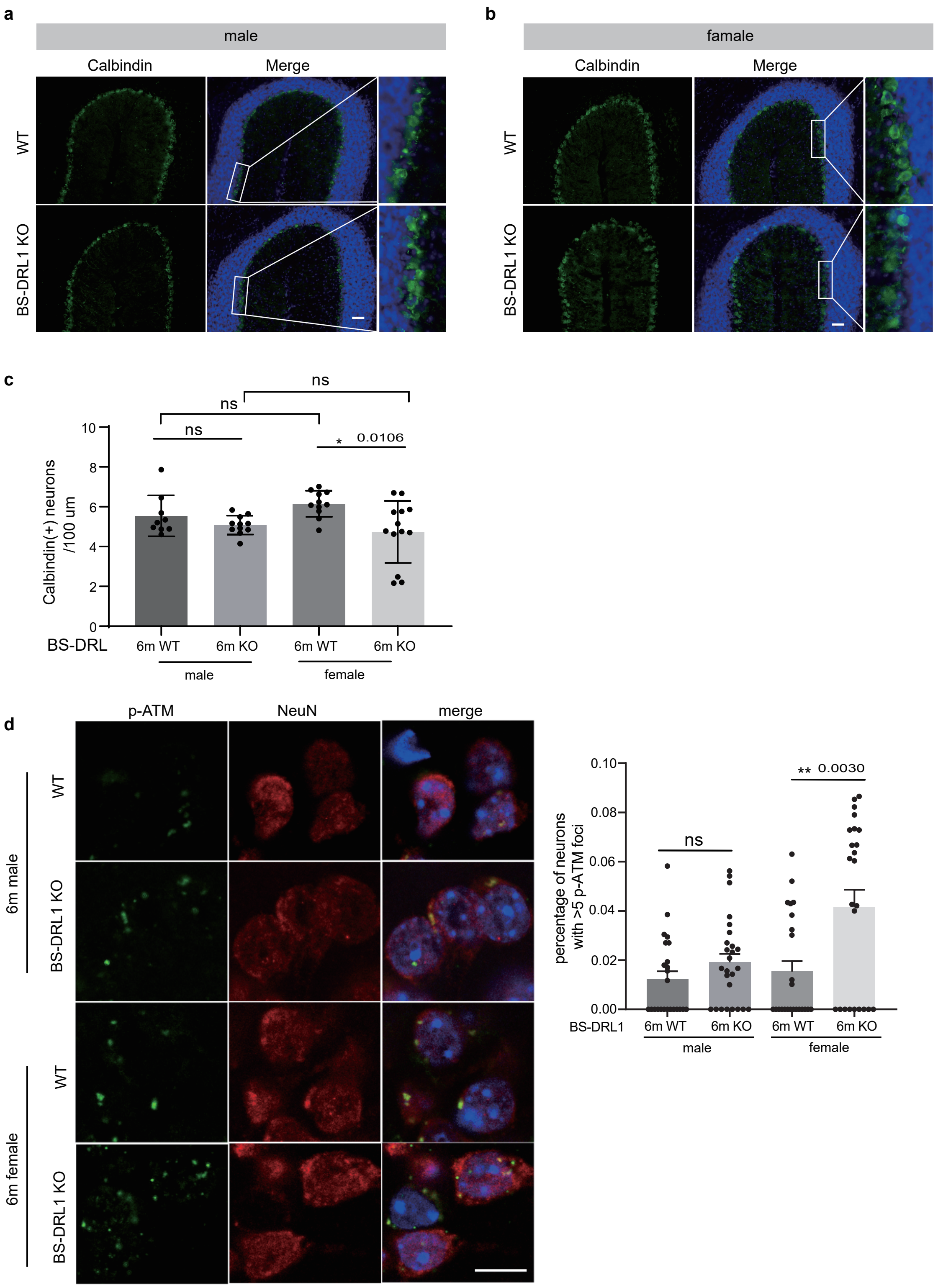

Supplementary Figure9. 6-month-old mice show slight degeneration on female.
a-c. Representative images showing immunostaining of purkinje cells (in green) and quantification. Immunofluorescence staining was performed with brain sections prepared from 6-month-old BS-DRL1 KO or littermate controls and calbindin antibody. Scale bar: 50 μm. Data are presented as mean ± SD. For each group, n=3. *p<0.05. ns: not significant.

d. p-ATM in Neurons of cerebral cortex sections from 6-month-old male and female BS-DRL1 KO mice or littermate controls. Scale bar: 10 μm. Data are presented as mean ± SD. For each group, more than 100 neurons were counted from 3 mice. **p<0.01. ns: not significant.

.

.

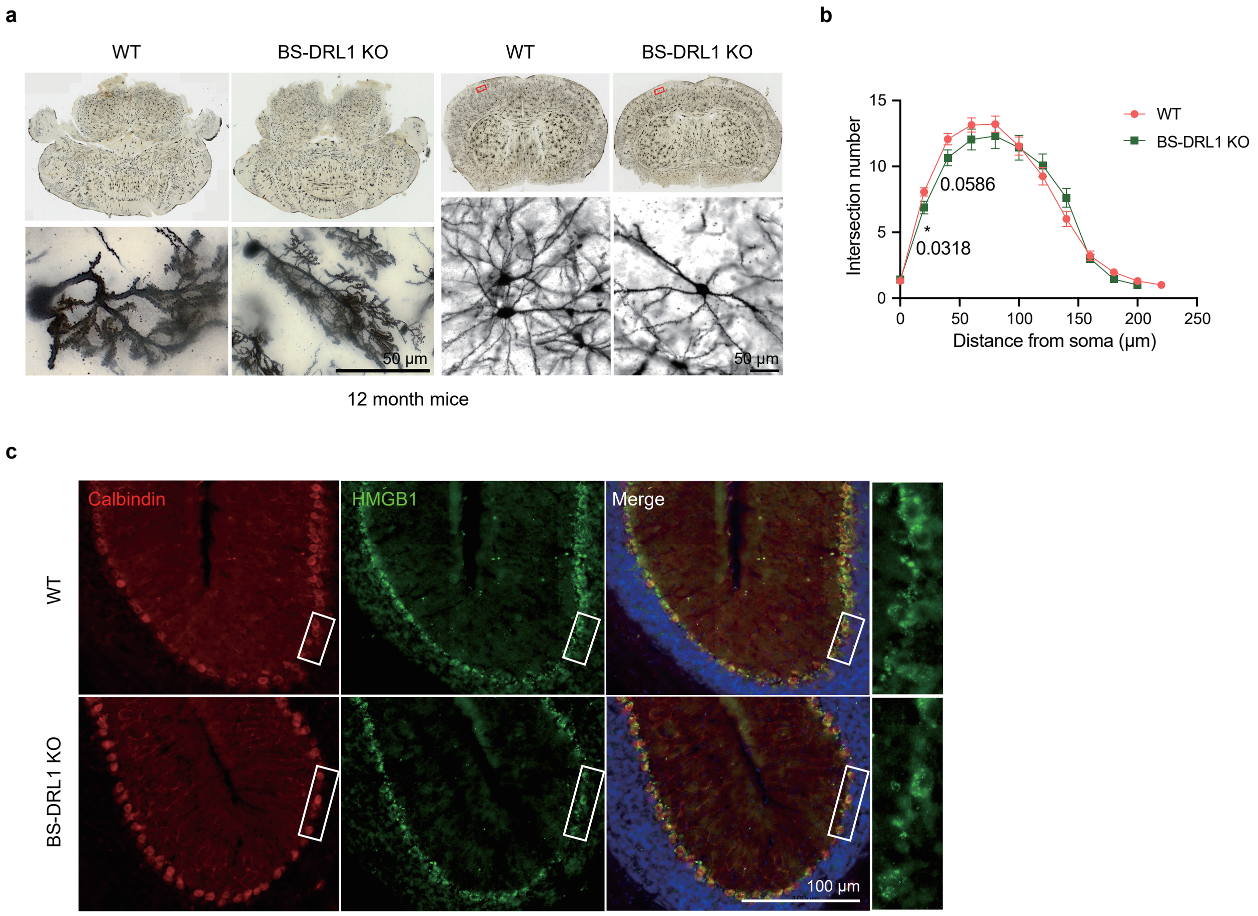

Supplementary Figure10. Neural cell morphology of BS-DRL1 KO mice.

a,b. Representative Golgi-Cox staining (FD Rapid Golgi Stain) in the cerebellum and cerebral cortex of 12-month-old BS-DRL1 KO mice or littermate controls. Purkinje cells had no difference between WT and BS-DRL1 KO mice. Cortex neuron as the red rectangle indicated were analyzed by sholl analysis using imageJ. Data are presented as mean ± SD, n=20 cells, *p<0.05.

c. HMGB1 expression level in purkinje cells was not affected by BS-DRL1. The brain sections were prepared from 12-month-old BS-DRL1 KO mice or littermate controls. n =3 biologically independent samples (mice).

a

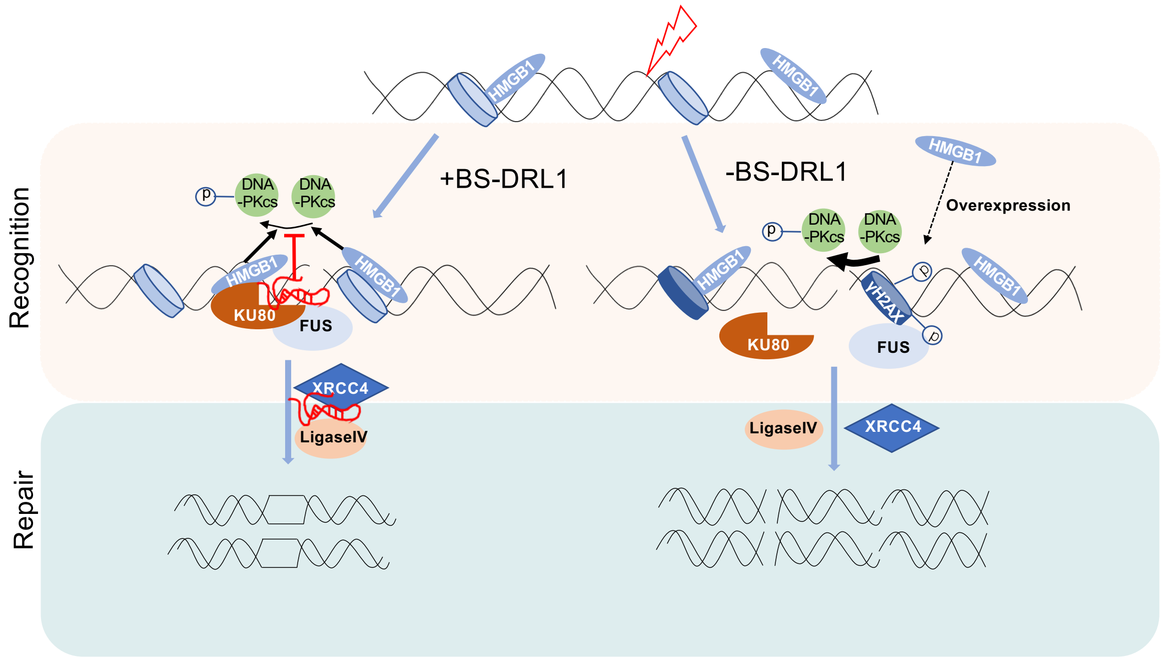

Supplementary Figure11. Working model for BS-DRL1 in DNA damage in neurons.

Schematic interpretation of BS-DRL1 in the protection of genomic stability in neurons. Upon DNA damage induction, BS-DRL1 can recruit HMGB1, KU80, et al to the damage sites to sense the DNA damage but to suppress the excessive response in the recognition step. In the next step, BS-DRL1 mediates the NHEJ components (e.g. XRCC4, LigaseIV) binding to damage sites to repair the damage to maintain the integrity of genomes. When BS-DRL1 is lost, the damage response is exaggerated (e.g. DNA-PKcs high phosphorylation), but the repair is defected, leading to instability of genomes.

a

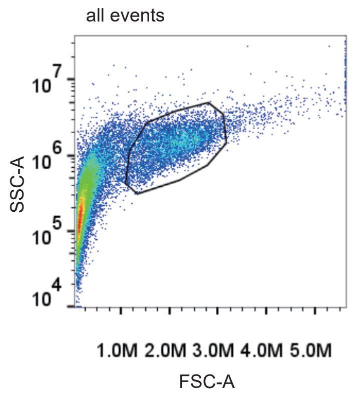

b

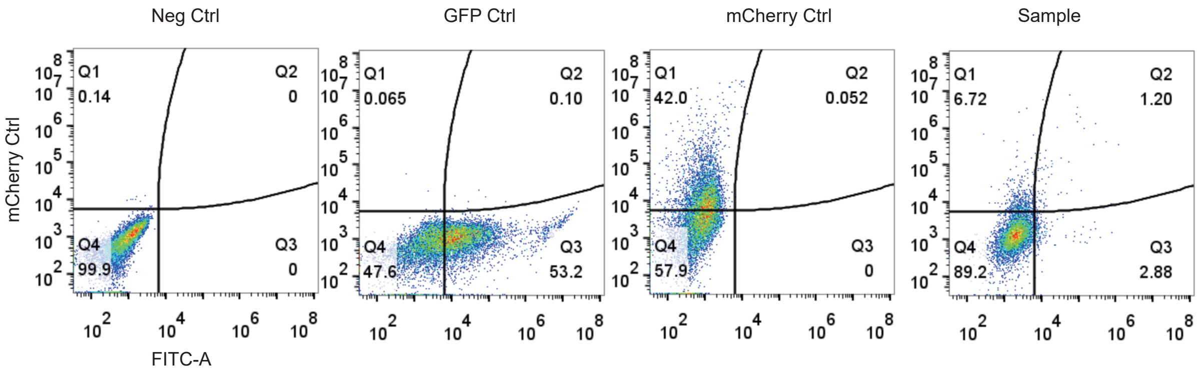

Supplementary Figure12. FACS sequential gating/sorting strategies.
Gating Strategies for cytometric analyses. (A) All events were gated for forward (FSC-A; approximate of size) and sideward scatter (SSC-A; approximate of granularity) to exclude debris. (B) Cells from (A) were gated for analysis of GFP (FITC) or mCHERRY positive cells in the sample. GFP or mCHERRY were gated on negative control (Neg Ctrl), and samples were further gated on GFP and mCHERRY control (GFP Ctrl, mCHERRY Ctrl). GFP+ cells were counted on Q2+Q3, mCHERRY+ cells were counted on Q1+Q2, the repair efficiency were counted as GFP+/mCHERRY+, i.e. (Q2+Q3)/(Q1+Q2). The used software was FlowJo X 10.0.7r2.
