# Supplementary material for: Long noncoding RNA BS-DRL1 modulates the DNA damage response and genome stability by interacting with HMGB1 in neurons
Source: Nat Commun. 2021 Jul 1;12:4075. doi: 10.1038/s41467-021-24236-z (PMC8249382; doi:10.1038/s41467-021-24236-z)
Supplement: Supplementary file 2 — Reporting Summary [file 41467_2021_24236_MOESM2_ESM.pdf]

nature research

Corresponding author(s): Wen-Yuan Wang

Last updated by author(s): Apr 22, 2021

Reporting Summary

Nature Research wishes to improve the reproducibility of the work that we publish. This form provides structure for consistency and transparency in reporting. For further information on Nature Research policies, see Authors & Referees and the Editorial Policy Checklist .

Statistics

For all statistical analyses, confirm that the following items are present in the figure legend, table legend, main text, or Methods section.

n/a	Confirmed	
☐	☒	The exact sample size (*n*) for each experimental group/condition, given as a discrete number and unit of measurement
☐	☒	A statement on whether measurements were taken from distinct samples or whether the same sample was measured repeatedly
☐	☒	The statistical test(s) used AND whether they are one- or two-sided<br>*Only common tests should be described solely by name; describe more complex techniques in the Methods section.*
☒	☐	A description of all covariates tested
☒	☐	A description of any assumptions or corrections, such as tests of normality and adjustment for multiple comparisons
☐	☒	A full description of the statistical parameters including central tendency (e.g. means) or other basic estimates (e.g. regression coefficient) AND variation (e.g. standard deviation) or associated estimates of uncertainty (e.g. confidence intervals)
☐	☒	For null hypothesis testing, the test statistic (e.g. *F*, *t*, *r*) with confidence intervals, effect sizes, degrees of freedom and *P* value noted<br>*Give P values as exact values whenever suitable.*
☒	☐	For Bayesian analysis, information on the choice of priors and Markov chain Monte Carlo settings
☒	☐	For hierarchical and complex designs, identification of the appropriate level for tests and full reporting of outcomes
☒	☐	Estimates of effect sizes (e.g. Cohen's *d*, Pearson's *r*), indicating how they were calculated

Our web collection on statistics for biologists contains articles on many of the points above.

Software and code

Policy information about availability of computer code

Data collection	we used Leica SP8 confocal microscope,Lecia ICC50 HD DMIL fluorescence microscope and Olympus vs200 for all images;<br>QuantStudio 6 Flex Real-Time PCR System for qPCR data;<br>Illumina HiSeq 10 with 2x150 paired-end sequencing for WGS in WuXi NextCODE at Shanghai<br>Thermo Scientific Orbitrap Fusion Tribrid for LC-MS/MS spectra .<br>ChIRP Probe Designer software (https://www.biosearchtech.com/support/tools/design-software/chirp-probe-designer
Data analysis	We used ImageJ for image(exclude comet assay image) analysis; CaspLab software for comet assay image; Prism for data summary ;<br>FlowJo software(X 10.0.7r2) for FACS data.<br>Burrows-Wheeler Aligner(BWA),Sentieon, CNVkit, Manta for WGS analysis;<br>Proteome Discover for Mass spectrometry data analysis.

For manuscripts utilizing custom algorithms or software that are central to the research but not yet described in published literature, software must be made available to editors/reviewers. We strongly encourage code deposition in a community repository (e.g. GitHub). See the Nature Research guidelines for submitting code & software for further information.

Data

Policy information about availability of data

All manuscripts must include a data availability statement. This statement should provide the following information, where applicable:

- Accession codes, unique identifiers, or web links for publicly available datasets
- A list of figures that have associated raw data
- A description of any restrictions on data availability

The data that support the findings of this study are available from the authors on reasonable request.
The analysis of active histone marks on the BS-DRL1 gene locus (Figure S1 f) was perform with public dataset (accession code: GSE29184).

Field-specific reporting

Please select the one below that is the best fit for your research. If you are not sure, read the appropriate sections before making your selection.

- [x] Life sciences
- [ ] Behavioural & social sciences
- [ ] Ecological, evolutionary & environmental sciences

For a reference copy of the document with all sections, see nature.com/documents/nr-reporting-summary-flat.pdf

Life sciences study design

All studies must disclose on these points even when the disclosure is negative.

Sample size	No sample size calculations were performed. Generally,experiments described in this study were performed with at least 3-15 samples for each group in accordance with community standards.
Data exclusions	No data were excluded from analysis.
Replication	3 independent biological replicates for in vitro analysis and 3 mice for immunofluorescence staining and at least 7 mice for mice behaviour tests. All attempts at replication were successful.
Randomization	Not relevant to our study since the experiments designed in our study were well controlled. i.e. WT vs KD or KO or OE; treatment vs no treatment
Blinding	The investigator were blind to the mice information.

Reporting for specific materials, systems and methods

We require information from authors about some types of materials, experimental systems and methods used in many studies. Here, indicate whether each material, system or method listed is relevant to your study. If you are not sure if a list item applies to your research, read the appropriate section before selecting a response.

Materials & experimental systems

n/a	Involved in the study	
[ ]	[x]	Antibodies
[ ]	[x]	Eukaryotic cell lines
[x]	[ ]	Palaeontology
[ ]	[x]	Animals and other organisms
[x]	[ ]	Human research participants
[x]	[ ]	Clinical data

Methods

n/a	Involved in the study	
[x]	[ ]	ChIP-seq
[ ]	[x]	Flow cytometry
[x]	[ ]	MRI-based neuroimaging

Antibodies

Antibodies used

Westerning blotting

Antibody	Species	D ilution	Cat. No.	Manufacturer
γH2AX	Mouse	3: 4000	05-636	Millipore
HMGB1	Rabbit	1: 1000	ab18256	Abcam
GAPDH	Mouse	1: 5000	60004-1-Ig	Proteintech
KU80	Mouse	1: 500	sc-515736	Santa Cruz
FUS	Mouse	1: 1000	sc-47711	Santa Cruz
XRCC4	Mouse	1: 500	sc-365055	Santa Cruz
H4	Mouse	1: 1000	2935	CST

ATM Mouse 1:1000 ab78 Abcam
p-ATM Mouse 1:1000 05-740 Millipore
DNA-PKcs Mouse 1:1000 ab1832 Abcam
p-DNA-PKcs Rabbit 1:1000 ab18192 Abcam
ACTIN Mouse 1: 10000 60008-1-Ig Proteintech
Immunofluorescence
Antibody Species Dilution Cat. No. Manufacturer
γH2AX Mouse 1: 500 0 5-636 Millipore
HMGB1 Rabbit 1: 500 ab18256 Abcam
Immunohistochemistry
Antibody Species Dilution Cat. No. Manufacturer
γH2AX Rabbit 1: 400 9718 CST
NeuN Chicken 1: 5000 ABN91 Millipore
53BP1 Rabbit 1: 2000 NB100-304 Novus Biologicals
HP1α Rabbit 1: 200 2616 CST
ATM Mouse 1:500 ab78 Abcam
p-ATM Mouse 1:500 05-740 Millipore
DNA-PKcs Mouse 1:500 ab1832 Abcam
p-DNA-PKcs Rabbit 1:500 ab18192 Abcam
Parvalbumin Mouse 1:5000 235 SWANT
Secondary antibody
Antibody Species Dilution Cat.No. Manufacturer
Goat anti-Rabbit IgG (H+L) Cross-Adsorbed Secondary Antibody, Alexa Fluor 488
Rabbit 1:1000 A-11008 Invitrogen
Goat Anti-Mouse IgG H&L (DyLight® 488)
mouse 1:1000 ab96879 abcam
Donkey Anti-Rabbit IgG H&L (Alexa Fluor® 568
Rabbit 1:1000 ab175470 abcam
Goat anti-Chicken IgY (H+L) Secondary Antibody, Alexa Fluor 568
Chicken 1:1000 A-11041 Invitrogen
Goat anti-Mouse IgG (H+L) Cross-Adsorbed Secondary Antibody, Alexa Fluor 568
mouse 1:1000 A-11004 Invitrogen
Goat Anti-Rabbit IgG H&L (Alexa Fluor® 594)
Rabbit 1:1000 ab150080 abcam
Goat Anti-Mouse IgG H&L (DyLight® 594)
mouse 1:1000 ab96873 abcam
Alexa Fluor® 647 AffiniPure Goat Anti-Rabbit IgG (H+L)
Rabbit 1:1000 111-605-003 Jackson ImmunoResearch

Validation

The antibodies are well validated for the indicated use by the manufacturer available on their websites.
Antibody Species Cat. No. supplier clone Cross reactivity Tested applications
53BP1 Rabbit NB100-304 Novus Biologicals Mouse, Rat, Human WB, Flow Cyt, IB, ICC/IF, IHC
ACTIN Mouse 60008-1-Ig Proteintech 7D2C10 Human, Mouse, Rat, Pig, Plant, Zebrafish Flow Cyt, IF, IHC, WB,ELISA
ATM Mouse ab78 Abcam 2C1 (1A1) Mouse, Rat, Human, Monkey Flow Cyt, ICC/IF, IHC-P, WB, IP
DNA-PKcs Mouse ab1832 Abcam 18-2 Mouse,Rat, Human, Common marmoset IHC-P, IHC-Fr, IP, WB, ICC/IF, Flow Cyt
DNA Ligase IV Rabbit ab80514 Abcam Mouse, Human WB, ICC/IF, IHC-P
FUS Mouse sc-47711 Santa Cruz 4H11 Mouse, Rat, Human WB, IP, IF, IHC
GAPDH Mouse 60004-1-Ig Proteintech 1E6D9 Human, Mouse, Rat, Plant, Yeast Flow Cyt, IF, IP, WB,ELISA
H4 Mouse #2935 CST L64C1 Mouse, Rat, Human, Monkey WB, IHC
HMGB1 Rabbit ab18256 Abcam Mouse, Rat, Human ICC/IF, WB
HP1α Rabbit #2616 CST Human, Mouse, Rat, Monkey Flow Cyt, ICC/IF, IHC-P, WB, IP
KU80 Mouse sc-515736 Santa Cruz B-4 Mouse, Rat, Human WB, IP, IF, IHC
NeuN Chicken ABN91 Millipore Mouse, Rat, Human (predicted) IHC, WB, ICC
p-ATM Mouse 05-740 Millipore 10H11.E12 Mouse, Human WB, IP, ICC
p-DNA-PKcs Rabbit ab18192 Abcam Human, Mouse (predicted) Flow Cyt, IHC-P, WB, ChIP, ELISA, ICC/IF
XRCC4 Mouse sc-365055 Santa Cruz A-7 Mouse, Rat, Human WB, IP, IF

γH2AX	Mouse	05-636	Millipore	JBW301	Vertebrates	ICC, IF, WB, ChIP, IHC
γH2AX	Rabbit	#9718	CST	20E3	Mouse, Rat, Human, Monkey	WB, IHC-P, IF, Flow Cyt
Parvalbumin	Mouse	#235	SWANT		Human, Monkey, Rabbit, Rat, Mouse, Chicken, Fish	IB, IHC

Eukaryotic cell lines

Policy information about cell lines

Cell line source(s)	HEK-293T () cell line were purchased from ATCC, USA.
Authentication	HKE-293T have been authenticated by STR method.
Mycoplasma contamination	The cell lines are negative for mycoplasma contamination
Commonly misidentified lines (See ICLAC register)	No commonly misidentified cell lines were used.

Animals and other organisms

Policy information about studies involving animals; ARRIVE guidelines recommended for reporting animal research

Laboratory animals	1. BS-DRL1 knockout mice were generated using CRISPR/Cas9 system on a C57BL/6N background by Beijing Biocytogen Co., Ltd., Beijing, China. P0-12 month old WT and BS-DRL1 KO male and female mice were maintained according to protocols approved by the Institutional Animal Care and Use Committee (IACUC) from Interdisciplinary Research Center on Biology and Chemistry (IRCBC), Shanghai Institute of Organic Chemistry, Chinese Academy of Sciences.<br>2.The E16 Pregnant ICR mice(8-10 weeks) used in this paper were purchased from Lingchang Company, Shanghai
Wild animals	none
Field-collected samples	none
Ethics oversight	All animal experiments were conducted according to protocols approved by the Institutional Animal Care and Use Committee (IACUC) from Interdisciplinary Research Center on Biology and Chemistry (IRCBC), Shanghai Institute of Organic Chemistry, Chinese Academy of Sciences.

Note that full information on the approval of the study protocol must also be provided in the manuscript.

Flow Cytometry

Plots

Confirm that:

- [x] The axis labels state the marker and fluorochrome used (e.g. CD4-FITC).
- [x] The axis scales are clearly visible. Include numbers along axes only for bottom left plot of group (a 'group' is an analysis of identical markers).
- [x] All plots are contour plots with outliers or pseudocolor plots.
- [x] A numerical value for number of cells or percentage (with statistics) is provided.

Methodology

Sample preparation	Primary neurons were infected with virus expressing indicated shRNAs after plating for 24h, and the cells were transfected with NHEJ plasmid for 2h after shRNAs were expressed for 48h, and changed the medium containing vehicle or etoposide for 1h, and the FACS analysis was performed 48h after treatment.
Instrument	CytoFLEX LX
Software	FlowJo VX X 10.0.7r2
Cell population abundance	Cells were derived from E16 ICR mice with high purity according to well validated protocol. GFP/mCherry positive cells were determined by the gating based on negative control.

Gating strategy	Cells were FSC/SSC gated firstly, then negative control without transfection and single transfection of GFP or mCherry were applied for the gating of cotransfection of GFP and mCherry.

☒ Tick this box to confirm that a figure exemplifying the gating strategy is provided in the Supplementary Information.
